# *Clostridium innocuum*, an opportunistic gut pathogen, inactivates host gut progesterone and arrests ovarian follicular development

**DOI:** 10.1080/19490976.2024.2424911

**Published:** 2024-11-07

**Authors:** Mei-Jou Chen, Chia-Hung Chou, Tsun-Hsien Hsiao, Tien-Yu Wu, Chi-Ying Li, Yi-Lung Chen, Kuang-Han Chao, Tzong-Huei Lee, Ronnie G. Gicana, Chao-Jen Shih, Guo-Jie Brandon-Mong, Yi-Li Lai, Po-Ting Li, Yu-Lin Tseng, Po-Hsiang Wang, Yin-Ru Chiang

**Affiliations:** a Department of Obstetrics and Gynecology, National Taiwan University Hospital and College of Medicine, National Taiwan University, Taipei, Taiwan; b Livia Shan-Yu Wan Chair Professor of Obstetrics and Gynecology, National Taiwan University, Taipei, Taiwan; c School of Medicine, National Tsing Hua University, Hsinchu, Taiwan; d Biodiversity Research Center, Academia Sinica, Taipei, Taiwan; e Department of Microbiology, Soochow University, Taipei, Taiwan; f Department of Agricultural Chemistry, National Taiwan University, Taipei, Taiwan; g Institute of Fisheries Science, National Taiwan University, Taipei, Taiwan; h Bioresource Collection and Research Center, Food Industry Research and Development Institute, Hsinchu, Taiwan; i Graduate Institute of Environmental Engineering, National Central University, Taoyuan, Taiwan; j Department of Chemical and Materials Engineering, National Central University, Taoyuan, Taiwan

**Keywords:** *Clostridium innocuum*, epipregnanolone, gut microbiome, infertility, low progesterone bioavailability, mouse, neurosteroid, progesterone, oral progesterone supplement

## Abstract

\Levels of progesterone, an endogenous female hormone, increase after ovulation; progesterone is crucial in the luteal phase to maintain successful pregnancy and prevent early miscarriage. Both endogenous and exogenous progesterone are recycled between the liver and gut; thus, the gut microbiota regulate host progesterone levels by inhibiting enterohepatic progesterone circulation. Our data indicated *Clostridium innocuum* as a major species involved in gut progesterone metabolism in women with infertility. *C. innocuum* converts progesterone into the neurosteroid epipregnanolone (with negligible progestogenic activity). We purified and characterized the corresponding enzyme, namely NADPH-dependent 5β-dihydroprogesterone reductase, which is highly oxygen sensitive and whose corresponding genes are prevalent in *C. innocuum*. Moreover, *C. innocuum*–administered female C57BL/6 mice (aged 7 weeks) exhibited decreased plasma progesterone levels (~35%). *Clostridium*-specific antibiotics (metronidazole) restored low plasma progesterone levels in these mice. Furthermore, prolonged *C. innocuum* administration (12 weeks) arrested ovarian follicular development in female mice. Cytological and histological analyses indicated that *C. innocuum* may cause luteal phase insufficiency and affect menstrual regularity. Our findings suggest *C. innocuum* as a causal factor of progesterone resistance in women taking progesterone.

## GRAPHICAL ABSTRACT

**Figure:**
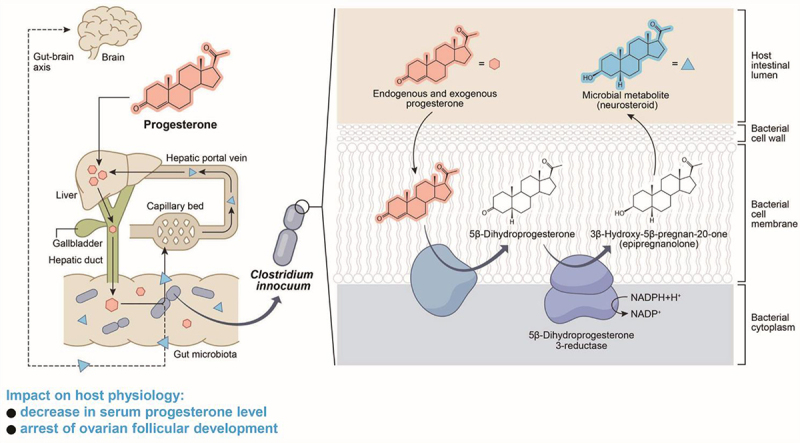

## Introduction

Reproductive hormones, including progestogens (see Figure S1 for the common structures of progestogens) and estrogens, modulate the metabolism, development, reproduction, and behavior of humans and other vertebrates.^1^ Progesterone plays a crucial role in fertilization, implantation, and embryogenesis in women and female mice.^2^ Progesterone has crucial functions in preparing the uterus for pregnancy and in maintaining pregnancy. Specifically, progesterone prepares the endometrium for implantation. In women, progesterone is mainly produced by the corpus luteum in the ovaries through two enzymatic steps; in the first step, the cholesterol side-chain cleavage enzyme (P450scc) transforms cholesterol into pregnenolone in the mitochondria, and in the second step, the human enzyme 3β-hydroxysteroid dehydrogenase converts pregnenolone into progesterone.^3^ Progesterone is primarily metabolized in the liver by the enzymes 5α-reductase and 3α-hydroxysteroid oxidoreductase, generating a series of metabolites [i.e., 3α-hydroxy-5α-pregnan-20-one (allopregnanolone)]. Allopregnanolone has protective and mood-stabilizing effects during pregnancy and the postpartum period.^4,5^ Progesterone and its metabolites are highly lipophilic and easily pass through the blood–brain barrier. These compounds thus accumulate in the brain and are considered neurosteroids.^6,7^ Previous studies have suggested that progesterone-derived neurosteroids, including allopregnanolone, are produced exclusively using human enzymes.^3^

In women, progesterone is present only during the luteal phase of the menstrual cycle. During pregnancy, blood progesterone levels are in the nanomolar range.^3^ Before childbirth, maternal progesterone levels may reach 200–2,000 nmol/L.^8^ Insufficient progesterone levels can (*i*) lead to luteal phase deficiency, affecting fertility and increasing the risk of miscarriage, and (*ii*) affect embryo implantation and increase the risk of uterine endometrial pathologies.^3^ For example, in women with chronic anovulation disorders such as polycystic ovary syndrome, insufficient progesterone levels are one of the most common causes of infertility and early miscarriage.^9–11^ Adequate progesterone supplementation in the luteal phase is essential to support successful embryo implantation and to prevent early miscarriage during fresh and frozen embryo transfer for women undergoing *in vitro* fertilization.

The bioavailability of the supplemented progesterone varies greatly among individuals and is often quite low (approximately 10%).^12^ In the first-pass effect, progesterone metabolism by hepatic enzymes is a major mechanism of low bioavailability. However, more than half of a sample of progesterone may be metabolized extrahepatically.^13^ Steroid hormones are recycled between the liver and gut through enterohepatic circulation. The reabsorption of steroids mainly occurs in the small intestine in humans^14^ and in the cecum in mice.^15,16^ Recent studies have indicated the crucial role of gut microbes in the modification and reabsorption of androgens in the gut.^17^ Similarly, circulating progesterone, derived from either endogenous production or exogenous administration, has been reported to be associated with the gut microbiota composition.^18–20^ However, the microbial species and corresponding enzymes responsible for progesterone metabolism in the gut remain unknown.

*Clostridium innocuum* is a Gram-positive, spore-forming anaerobe that significantly affects the metabolism and functioning of the gastrointestinal tract in humans. A comparative genomic analysis of publicly available microbiota datasets related to healthy individuals indicated a high prevalence of *C. innocuum* in the global population (>80%).^21^ Despite being previously recognized as a commensal bacterium, *C. innocuum* has recently been associated with multiple health conditions, including diarrhea, bacteremia, endocarditis, osteomyelitis, and peritonitis.^22–25^ This finding suggests that *C. innocuum* is an opportunistic pathogen; however, a related virulence mechanism is yet to be identified. Previous studies have indicated that certain inflammatory diseases (such as diarrhea- and bacteremia-related endotoxemia) are associated with disturbed progesterone metabolism.^26,27^ However, the role of the human microbiome in these bioprocesses remains to be elucidated. In the present study, we identified *C. innocuum* as a major species responsible for gut progesterone metabolism. Moreover, we applied integrated omics approaches to identify the progestogenic metabolites, microbial enzymes, and corresponding genes. The oral administration of *C. innocuum* to female mice led to substantial decreases in serum progesterone levels and the arrest of both the estrous cycle and ovarian follicular development.

## Results

### Anaerobic progesterone metabolism by the gut microbiota in female patients with infertility

Several studies have demonstrated that both endogenous progesterone and exogenous progesterone can alter the host gut microbiota. For example, progesterone treatment inhibited the germination of *Clostridium difficile* spores^28^ and led to the abundance of *Bifidobacterium*^19^ and *Lactobacillus*^29^ in the guts of female mice. Furthermore, progesterone has been reported to be metabolized by gut microbes. Kornman and Loesche^29^ demonstrated that *Bacteroides* species likely utilized progesterone for their growth. Therefore, we hypothesized that female patients with infertility may harbor progesterone-metabolizing microbes that utilize progesterone in the gut and disrupt the enterohepatic circulation of progesterone. To validate this hypothesis, we collected fresh fecal samples from 14 female patients with infertility (aged 20–40 years; see Table 1 for detailed information) who received oral progesterone administration for endometrial preparation and thawed embryo transfer.

**Table 1.** Physiological characteristics of 14 women with infertility.

Patient No.	Progesterone metabolic activity*		Age (years)	BMI (kg/m^2^)	Indication for IVF	Menstrual regularity	AMH level (ng/ml)	Pregnancy outcome
Case 1		active	36.2	36.7	Primary infertility and tubal factor	irregular	1.76	live birth
Case 2		inactive	35.9	30.9	Primary infertility	irregular	6.52	chemical pregnancy
Case 3		inactive	36.8	27.2	Secondary infertility	regular	NA	chemical pregnancy
Case 4		inactive	33.3	24.3	Primary infertility and male factor	regular	4.82	live birth
Case 5		inactive	35.4	20.5	Primary infertility	regular	4.03	no pregnancy
Case 6		inactive	35.9	21.7	Primary infertility and male factor	regular	1.31	no pregnancy
Case 7		active	38.9	31.3	Primary infertility	regular	0.62	chemical pregnancy
Case 8		inactive	34.5	21.1	Primary infertility and premature ovarian failure	irregular	<0.02	live birth
Case 9		inactive	37.4	21.6	Primary infertility and tubal factor	regular	3.78	no pregnancy
Case 10		active	37.4	19.2	Secondary infertility	irregular	NA	chemical pregnancy
Case 11		active	32.2	20.3	Secondary infertility and tubal factor	regular	2.65	chemical pregnancy
Case 12		inactive	42.6	22.5	Primary infertility and tubal factor	regular	2.57	live birth
Case 13		active	40.4	23.8	Secondary infertility and male factor	regular	4.38	live birth
Case 14		active	40.4	39.5	Primary infertility and tubal factor	irregular	6.38	live birth

NA: not available.

*Progesterone metabolic activity was determined by quantifying residual progesterone remaining in fecal cultures that were anaerobically incubated with progesterone (1 mm) in DCB-1 medium for 7 days. “Active” is represented by microbial utilization of >50% of progesterone molecules.

**Figure 2.**
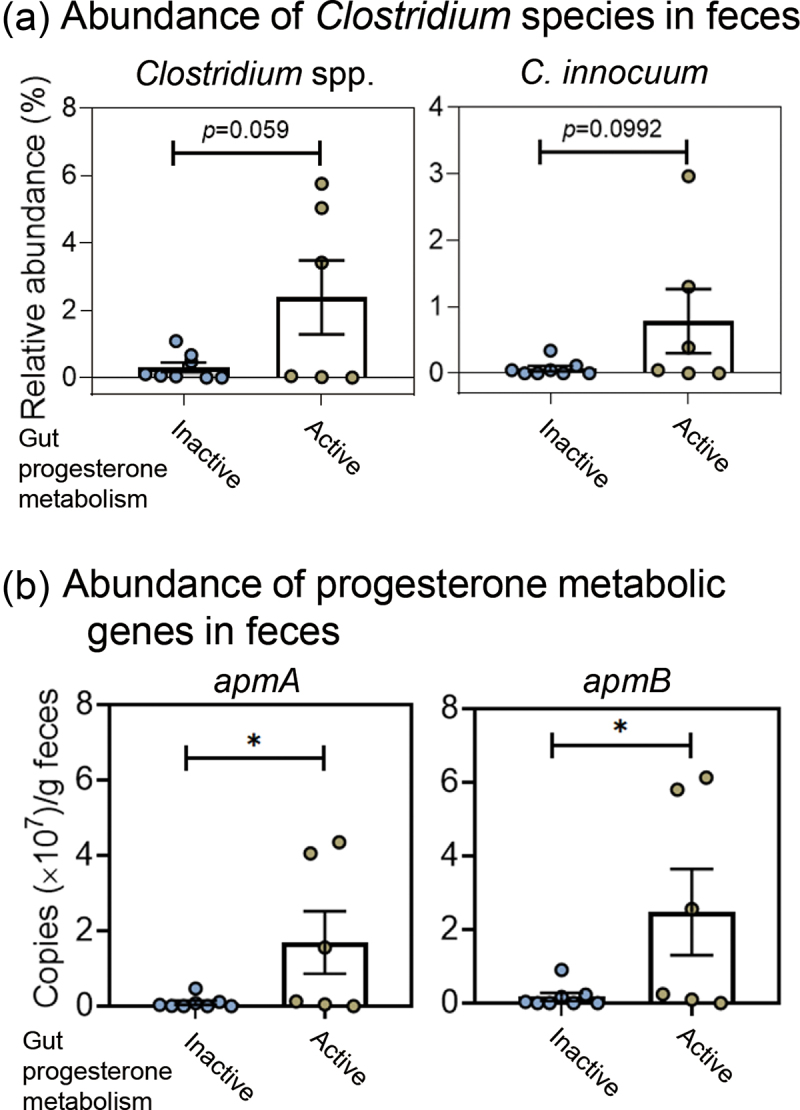
Differences in the abundance of progesterone-metabolizing gut microbes and their corresponding genes between patients exhibiting active and inactive gut progesterone metabolism. Detailed information regarding the physiological characteristics of 14 patients is provided in Table 1. (a) Relative abundance (%) of the bacterial genus *Clostridium* and the *C. innocuum* species in fresh fecal samples collected from 14 patients. (b) Abundance of the progesterone metabolism genes *apmAB* in the fresh fecal samples. Statistical results were calculated with unpaired nonparametric *t*-test; *p < 0.05.

Previous gut microbiome studies have shown that certain *Clostridium* species may metabolize progesterone.^3,20,28^ This study investigated whether *Clostridium* spp. are highly abundant in the gut microbiota of infertile patients showing strong progesterone metabolic activity (see Table 1 for demographic and hormonal characteristics). We categorized participants into two groups based on the progesterone metabolic activity of their gut microbiota: an Inactive group (*n* = 8) with negligible activity and an Active group (*n* = 6) with observable activity (Table 1). Analysis of fresh fecal samples revealed a trend toward higher abundance of the genus *Clostridium* in the Active group (*p* = 0.059; Figure 2(a), left panel). Furthermore, we observed a marginally higher abundance of *C. innocuum* in the Active group (*p* = 0.099; Figure 2(a), right panel). However, we did not detect apparent difference in the abundance of other *Clostridium* species between the two groups.

**Figure 3.**
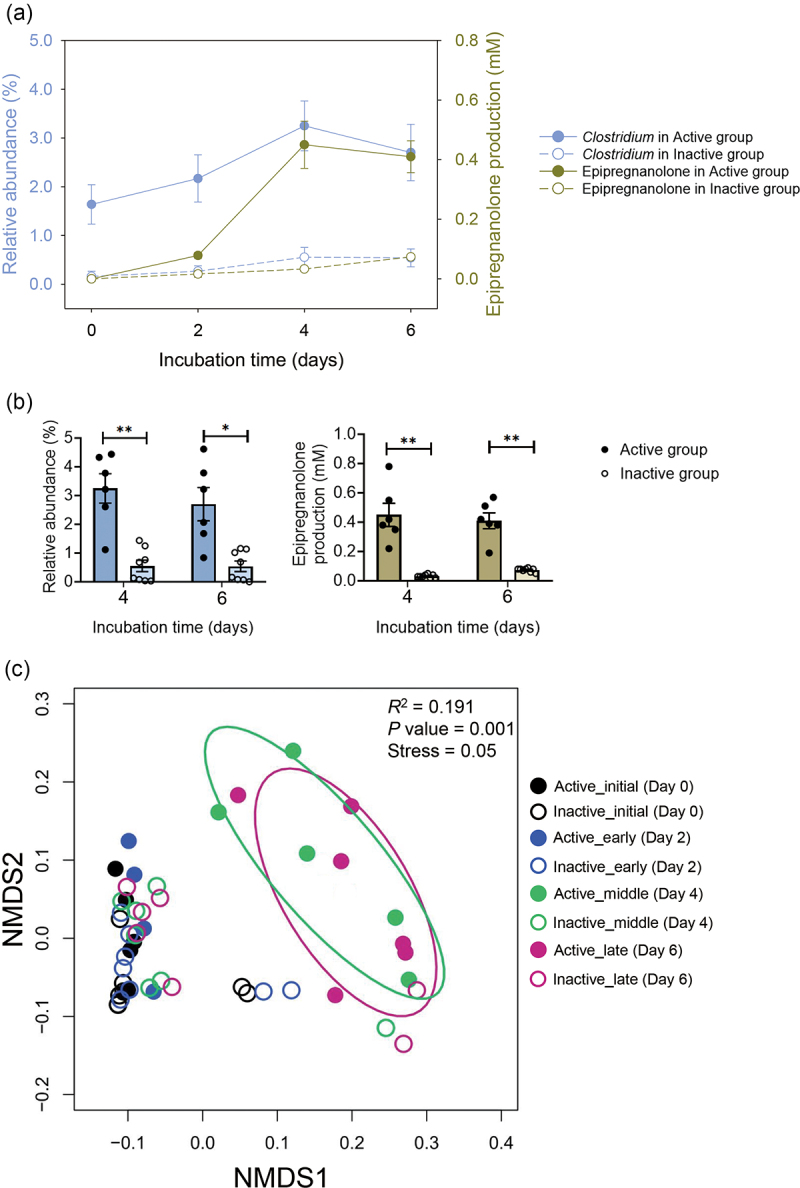
*Clostridium* growth and epipregnanolone production in the progesterone-amended fecal cultures. Fresh fecal samples from 14 patients were anaerobically incubated in the DCB-1 medium containing progesterone (1 mm) for 6 days. (a) The growth of *clostridium* and the production of epipregnanolone in the active (*n* = 6) and inactive (*n* = 8) fecal cultures amended with progesterone. (b) *Clostridium* abundance and epipregnanolone production is significantly higher in the progesterone-treated active gut microbiota with longer incubation duration (≥4 days). Data shown in a and b are mean ± standard error for 6 (active group) and 8 (inactive group) individuals. Statistical results were calculated with unpaired nonparametric *t*-test; *p < 0.05, **p < 0.01. (c) The NMDS analysis of the progesterone-treated gut microbiota. Fresh fecal samples collected from a total of 14 female patients (active group, *n* = 6; inactive group, *n* = 8) were anaerobically incubated in the DCB-1 broth containing 1 mm of progesterone for 6 days, and the fecal cultures were sampled at different incubation stages.

To further investigate the viability and progesterone metabolic activity of *Clostridium* species, we conducted an enrichment experiment. Fresh fecal samples from 14 patients were anaerobically incubated with progesterone (1 mm) in a chemically defined mineral medium (DCB-1)^30^ for 6 days, with sampling every 2 days. DNA extraction, bacterial 16S rRNA gene amplification, and PacBio sequencing were performed to analyze temporal changes in bacterial community structures. In the Active group (*n* = 6), we observed a strong correlation between the increase in *Clostridium* abundance and epipregnanolone production in the progesterone-amended fecal cultures (*R*^2^ = 0.81) (Figure 3(a)). Conversely, the Inactive group (*n* = 8) showed negligible increases in both *Clostridium* abundance and epipregnanolone production. The progesterone-enriched gut microbiota from the Active group exhibited a significantly higher abundance of the genus *Clostridium* at later incubation stages (≥4 days) (Figure 3(b), left panel), which is corresponded with the apparent production of epipregnanolone by the Active gut microbiota (Figure 3(b), right panel). We also employed non-metric multidimensional scaling (NMDS) to analyze the diversity and temporal changes in the progesterone-treated gut microbiota. The NMDS analysis considered 16S rRNA amplicons and the contents of progesterone (substrate) and epipregnanolone (major product) in cultural samples. Initial samples (Day 0) clustered together on the left-hand side of the coordinate, showing similar *Clostridium* abundance and steroid contents. Active gut microbiota samples from later incubation stages (Days 4 and 6) clustered separately at the top right of the coordinate, characterized by higher *Clostridium* relative abundance, progesterone consumption, and epipregnanolone content. Inactive gut microbiota samples from later stages remained grouped with initial samples or clustered separately at the bottom of the coordinate. Permutational multivariate analysis of variance (PERMANOVA) revealed that 19% of the data contributed to the significant difference (global *R*^2^ = 0.191; *p* value = 0.001) between the Active and Inactive groups (Figure 3(c)). Collectively, our data suggest a key role for *Clostridium* species, particularly *C. innocuum*, in gut progesterone metabolism.

To investigate whether *C. innocuum* could convert progesterone to epipregnanolone under anaerobic conditions, we isolated 25 *C. innocuum* strains from various female patients. We spread progesterone-enriched fecal cultures on Brain Heart Infusion (BHI) medium containing 2 mm progesterone. The resulting colonies were incubated with 1 mm progesterone, and microbial products were analyzed using ultraperformance liquid chromatography (UPLC)–atmosphere pressure chemical ionization (APCI)–high-resolution mass spectrometry (HRMS) (Table S1). Of the 25 *C. innocuum* strains, 18 were isolated from patients showing strong progesterone metabolic activity (Active group), with 5 strains from Patient 1 alone. Physiological tests revealed that all isolates metabolized progesterone, primarily producing epipregnanolone. Additionally, 14 strains produced pregnanolone, and 3 strains produced isopregnanolone as minor products (Table 2). Notably, none of the tested *C. innocuum* strains produced allopregnanolone.

**Table 2.** Isolation and characterization of *C. innocuum* strains from female patients with infertility. Patients with apparent progesterone metabolic activity are highlighted in bold.

Bacterial strains	Hosts	Progesterone Metabolic activity*	Major microbial products			
			Allo-pregnanolone	Iso-pregnanolone	Pregnanolone	Epi-pregnanolone
BT6	**Patient 1**	+	-	-	+	+
RBW1-1	**Patient 1**	++	-	-	+	+
RGG8	**Patient 1**	+++	-	-	+	+
BP16	**Patient 1**	+	-	-	+	+
RBW2-1	**Patient 1**	+	-	-	+	+
BT3-1	Patient 3	+	-	-	-	+
RBW3	Patient 3	+	-	-	-	+
RBW4-1	Patient 4	+	-	-	-	+
RBW5	Patient 5	+	-	+	-	+
RBW6-1	**Patient 7**	+	-	+	+	+
RBW6-2	**Patient 7**	+	-	-	+	+
BT16	**Patient 7**	++	-	-	-	+
RBW8-1	Patient 8	+	-	-	+	+
RBW8-3	Patient 8	+	-	-	-	+
RBW9-1	Patient 9	++	-	-	-	+
RBW9-3	**Patient 10**	+	-	+	+	+
L3E5	**Patient 10**	++	-	-	+	+
BP26	**Patient 10**	++	-	-	-	+
RBW11-1	**Patient 11**	++	-	-	-	+
RBW11-2	**Patient 11**	++	-	-	+	+
RBW13-1	**Patient 13**	++	-	-	+	+
RBW13-2	**Patient 13**	++	-	-	-	+
RBW13-3	**Patient 13**	++	-	-	+	+
RB14	**Pateint 14**	++	-	-	-	+
BP14	**Patient 14**	++	-	-	+	+

*(+) symbol indicates the activity of anaerobic progesterone metabolism. Bacterial strains were cultivated in the Gifu anaerobic medium (GAM) containing 2 mm of progesterone for 48 h. +++Over 80% of the substrate was consumed. ++ Over 50% (but less than 80%) of the substrate was consumed. +, Over 10% (but less than 50%) of the substrate was consumed.

**Figure 4.**
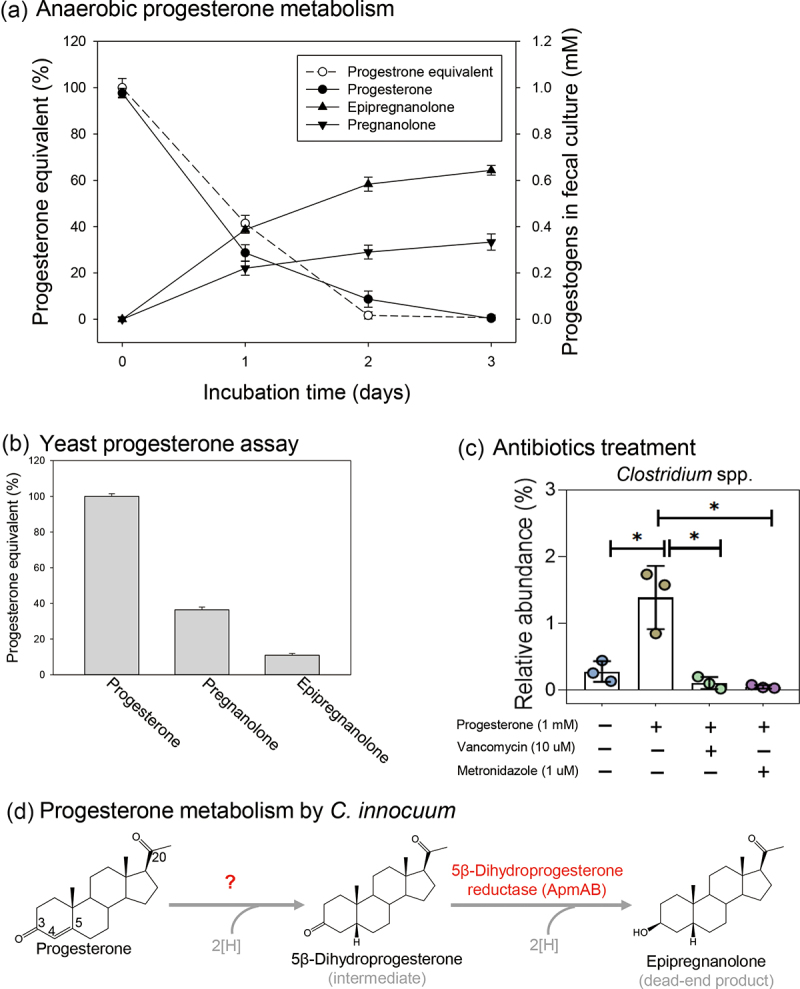
Identification of key players in gut progesterone metabolism and their corresponding genes in the gut microbiota of patient 1. (a) Time-course progesterone utilization, microbial metabolite production, and progestogenic activities in fecal cultures. (b) Relative progestogenic activities (%) of individual progestogens. The progestogenic activity was determined through the yeast progesterone assay. (c) Inhibition of *Clostridium* growth and progesterone metabolism by two *Clostridium*-specific antibiotics (see Figure S1C for the effects of individual antibiotics on progesterone metabolism). Data are expressed as mean ± standard error for three independent experiments. Statistical results were calculated with unpaired nonparametric *t*-test; *p < 0.05. (d) Progesterone metabolism products and the characterized progesterone metabolism enzymes of *C. innocuum*.

Among these patients, Patient 1 exhibited characteristic symptoms of chronic anovulation, obesity, and considerably low bioavailability of oral progesterone supplementation (Table 1). The fresh fecal sample from Patient 1 contained the most abundant *Clostridium* spp. (5.7%) and *C. innocuum* (3.0%) (Figure 2(a)). Moreover, the gut microbiota of Patient 1 exhibited strong progesterone metabolic activity (Figure 3 and Table 2). Therefore, we investigated the progesterone metabolic activity of the gut microbiota of Patient 1 at molecular levels. This patient’s fecal sample (approximately 0.5 g) was anaerobically incubated with progesterone (1 mm) in the chemically defined DCB-1 medium^30^ or in the BHI medium (a nutrient-rich medium). Progesterone-derived microbial products were extracted and identified through UPLC–APCI–HRMS. In progesterone (1 mm)-amended fecal cultures, we observed apparent progesterone utilization by the gut microbiota, which was consistent with the marked decrease in progestogenic activity observed over time (Figure 4(a)). Two progesterone-derived microbial products, epipregnanolone (3β-hydroxy-5β-pregnan-20-one; the major product) and pregnanolone (3α-hydroxy-5β-pregnan-20-one; the minor product), were observed in both the BHI (Figure 4(a)) and DCB-1 cultures (Figure S1Aii; left panel). We then determined the progestogenic activities of individual progestogens (i.e., progesterone, epipregnanolone, and pregnanolone) through a yeast progesterone assay, and the aforementioned two microbial products exhibited considerably low progestogenic activity (Figure 4(b)). Among the two products, epipregnanolone exhibited the lowest progestogenic activity (a decrease of 89% compared with progesterone). These data indicated that the fecal sample from Patient 1 harbored highly active gut microbes that were able to metabolize progesterone (with a 3-keto group) into 3α- or 3β-hydroxyl structures (the microbial products and enzymes characterized in this study are provided in Figure 4(d)), leading to a decrease in progestogenic activity.

### Identification and characterization of key gut microbes responsible for progesterone metabolism

We managed to identify major progesterone-metabolizing gut microbes. Sequencing on the PacBio platform revealed an increase in the abundance of the genus *Clostridium* in the fecal cultures of Patient 1 after anaerobic incubation with progesterone (1 mm) for 3 days (Figure S1B). We used different antibiotics to selectively inhibit specific populations in the gut microbiota. We observed that two first-line antibiotics typically applied for *Clostridium* infection treatment [vancomycin (10 μM) and metronidazole (1 μM)] inhibited the progesterone-caused enrichment of *Clostridium* population (Figure 4(c)). Subsequently, we investigated the effects of these antibiotics on anaerobic progesterone metabolism. First, we used two broad-spectrum antibiotics (tetracycline and thiamphenicol) to inhibit this microbial activity, and it was inhibited by a relatively high concentration of tetracycline (minimum inhibition concentration = 50 μM) and thiamphenicol (minimum inhibition concentration = 25 μM) (Figures S1Ci and S1Cii). We then used two *Clostridium*-specific antibiotics (vancomycin and metronidazole) to inhibit anaerobic progesterone metabolism in progesterone-amended fecal cultures. Progesterone metabolism was highly inhibited by metronidazole (minimum inhibition concentration = 1.5 μM) and moderately inhibited by vancomycin (minimum inhibition concentration = 10 μM) (Figures S1Ciii and S1Civ); these results indicated that *Clostridium* spp. likely are responsible for the anaerobic progesterone metabolism.

### *Isolation and characterization of* Clostridium *species from the gut microbiota*

We isolated *Clostridium* species from the gut microbiota of Patient 1. In total, we characterized 22 gut *Clostridium* strains belonging to seven different *Clostridium* species (Table 3). Of these 22 strains, only five (5/22; 23%) exhibited progesterone metabolic activity, and all of them were *C. innocuum* (Table 3). All these five *C. innocuum* strains produced epipregnanolone as the main product after anaerobic incubation with progesterone, whereas pregnanolone was produced only in trace amounts. Additionally, these *C. innocuum* strains were highly sensitive to metronidazole (with minimum inhibition concentration at 1.3 and 0.3 μg/mL in the BHI broth and BHI agar, respectively); by contrast, these strains were less sensitive to vancomycin, which was consistent with the results of the antibiotic inhibition trials (Figures 4(c) and S1C). Our data thus indicated the crucial role of *C. innocuum* in gut progesterone metabolism. Because the *C. innocuum* strain RGG8 exhibited the highest progesterone metabolic activity (Tables 2 and 3), we used the strain RGG8 as the model microorganism for subsequent genomic and enzymatic characterization.

**Table 3.** Isolation and characterization of *Clostridium* species from the fecal samples of patient 1.

Bacterial Identities		Progesterone transformation		progesterone metabolic genes		Antibiotics susceptibility#		
*Clostridium* species	Strains	Epipregnanolone production	Pregnanolone production	*apmA* (LMAICMKE_01819)	*apmB* (LMAICMKE_01820)	Metronidazole (broth) (MIC; μg/mL)	Metronidazole (agar) (MIC; μg/mL)	Vancomycin (broth) (MIC; μg/mL)
*C. innocuum*	RGG8	+++	+	+	+	1.25	0.30	>2.50
	RBW1-1	++	+	+	+	1.25	0.30	>2.50
	BT6	++	+	+	+	1.25	0.30	>2.50
	BP16	++	+	+	+	1.25	0.30	>2.50
	RBW2-1	++	+	+	+	1.25	0.30	>2.50
C. difficile*	BCRC17678	-	-	-	-	1.25	0.25	2.50
	BCRC17702	-	-	-	-	1.25	0.25	2.50
	BCRC17900	-	-	-	-	1.25	0.25	2.50
	BCRC80997	-	-	-	-	1.25	0.25	2.50
	BCRC80998	-	-	-	-	1.25	0.25	1.25
	BCRC80999	-	-	-	-	0.63	0.25	1.25
*C. butyricum*	M3	-	-	-	-	0.31	0.10	1.25
	C14-1	-	-	-	-	0.31	0.10	1.25
	C14-4	-	-	-	-	0.31	0.10	1.25
	C14-50	-	-	-	-	0.31	0.10	1.25
	C14-51	-	-	-	-	0.31	0.10	1.25
*C. scindens*	L1-10	-	-	-	-	0.15	0.70	2.50
	L1-28	-	-	-	-	0.15	0.70	2.50
	L1-33	-	-	-	-	0.15	0.70	2.50
*C. subterminale*	L2B4-2a	-	-	-	-	>2.50	>2.00	1.25
*C. tertium*	L2D7-4	-	-	-	-	>2.50	>2.00	1.25
*C. acetobutylicum*	L3D7-7	-	-	-	-	0.15	0.10	1.25

*Strains of *C. difficile* were obtained from Bioresource Collection and Research Center, Food Industry Research and Development Institute, Hsinchu, Taiwan, because the gut microbiota of Patient 1 did not contain *C. difficile*.

^#^The antibiotics were tested in the dose ranging from 0.02 to 2.50 μg/mL (for bacterial growth in broth) and 0.02 to 2.00 μg/mL (for bacterial growth on agar).

+++Over 80% of the substrate was transformed into corresponding product. ++ Over 50% (but less than 80%) of the substrate was transformed into corresponding products. +, Over 10% (but less than 50%) of the substrate was transformed into corresponding products.

### *Characterization of progesterone metabolism enzymes from* C. innocuum *strain RGG8*

Given that the reduction of the 3-keto group in progesterone significantly decreased its activity (Figure 4(b)), we purified and characterized the microbial enzyme responsible for the reduction of the 3-keto group. The resting cell assays revealed that the extracellular electron carrier methyl viologen and the strong reductant titanium citrate were required for the progesterone metabolism of *C. innocuum* (Figure S2A), which is a typical characteristic of the enzymatic reactions of O_2_-labile [4Fe-4S] proteins.^31,32^ Moreover, in the resting cell assays, the ATPase inhibitor (sodium orthovanadate) did not significantly reduce progesterone metabolic activity (Figure S2A); this observation implies that progesterone metabolism does not require ATP-dependent uptake to transport progesterone into the cytoplasm and that the enzymes responsible for the reduction of the 3-keto group are likely located on the cell membrane.

**Figure 5.**
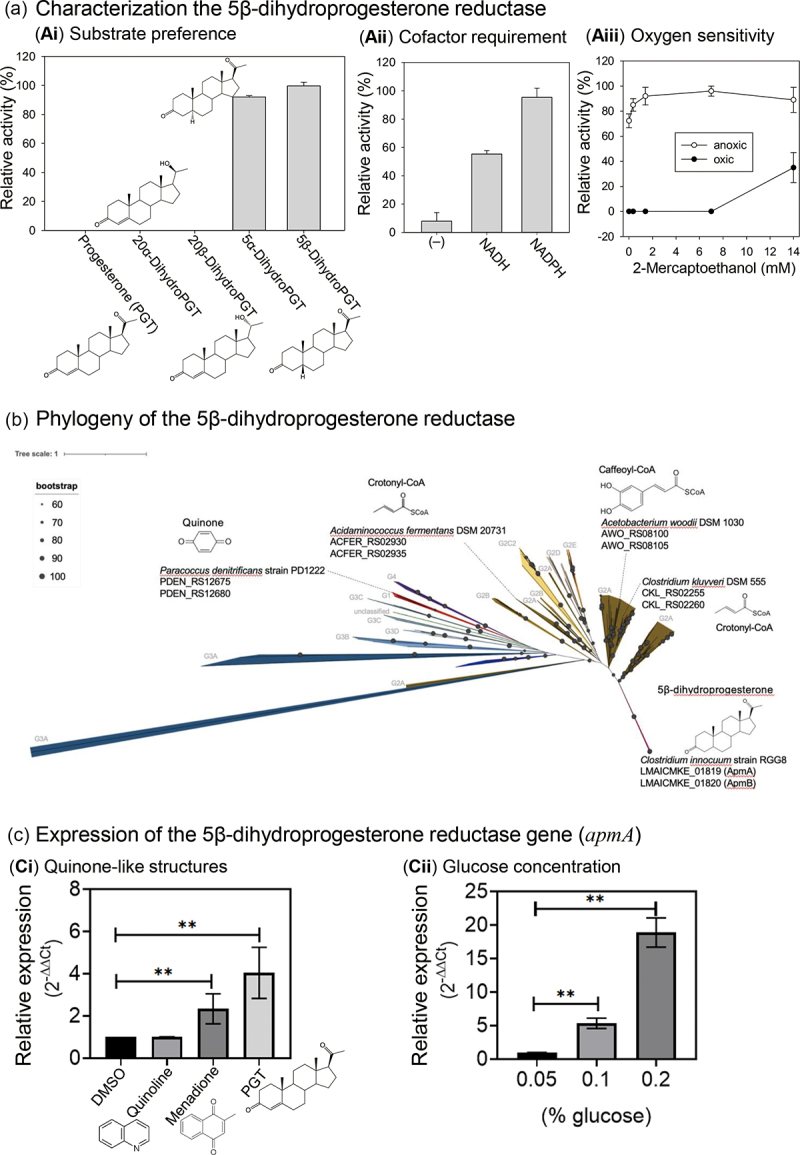
(a) Characterization of NADPH-dependent 5β-dihydroprogesterone reductase (ApmAB) purified from *C. innocuum* strain RGG8. (b) Phylogenetic analysis of 356 Etfαβ sequences, (including 206 ApmAB). The phylogenetic relationship was determined using Maximum-likelihood method and the bootstrap values were calculated based on 500 replicates. (c) Expression of *apmA* induced by progesterone (Ci) and glucose (Cii). All three cyclic compounds had a working concentration of 0.3 mm. The final DMSO concentration in the assay mixtures was 0.3% (v/v). Abbreviations: PGT, progesterone.

Through chromatographic separation, we purified and characterized the *C. innocuum* enzyme, likely a peripheral membrane protein, responsible for the anaerobic reduction of the 3-keto group. The purified enzyme utilized 5β-dihydroprogesterone as the optimal substrate and did not use progestogens with a double bond at C-4 (*e.g*., progesterone, 20α-dihydroprogesterone, and 20β-dihydroprogesterone) as the substrate (Figure 5Ai). With 5β-dihydroprogesterone serving as the substrate, the optimal pH of the purified protein was approximately pH 8.5 (Figure S2Bi), and the optimal working temperature was 25°C. Purified 5β-dihydroprogesterone reductase was still active (relative activity >60%) at 45°C (Figure S2Bii). For 5β-dihydroprogesterone reductase, NADPH was the preferred electron donor (Figure 5Aii). Additionally, this enzyme was highly sensitive to O_2_. Under aerobic conditions, the activity of 5β-dihydroprogesterone reductase was partially maintained with a high concentration of the reductant 2-mercaptoethanol (Figure 5Aiii).

Proteomics analysis of the closed genome of the strain RGG8 (accession no.: CP150439) revealed tryptic peptides originating from the active protein fraction match with the predicted tryptic products of strain RGG8 genes LMAICMKE_01819 (*apmA*) and LMAICMKE_01820 (*apmB*) (Dataset S1). Bioinformatic analysis indicated that ApmAB represented a new member of the electron-transferring flavoprotein (Etf) family, and the results suggested that both ApmA and ApmB contain the FAD-binding domain, whereas only the ApmA component possesses a [4Fe-4S] cluster as the prosthetic group (Dataset S1). In addition to ApmAB in the strain RGG8, we collected 205 ApmAB sequences (identity >98%) from publicly available *C. innocuum* genomes; using these data, we elucidated the phylogenetic relationship of ApmAB and other Etf members (150 sequences in total).^33^ We then constructed a phylogenetic tree with 356 concatenated Etfα and Etfβ protein sequences (Figure 5(b)). On the basis of this phylogenetic tree, we classified Etfαβ into five groups, which were similar to those reported by Costas et al.^33^ The ApmAB sequences formed a distinct subgroup within the G2 group. Noticeably, *apmAB* are prevalent in *C. innocuum*, and they have not been identified in other *Clostridium* species (Figure 5(b)). Given that *apmAB* are highly conserved among *C. innocuum* species, we designed specific primers for *apmA* and *apmB* (see Table S2 for nucleotide sequences). Our studies regarding the expression of the *apmA* (the [4Fe-4S]-containing component) revealed that this gene can be induced by progesterone and menadione (another quinone-like structure), albeit to a lesser extent (Figure 5ci). By contrast, the aromatic quinoline did not induce the expression of *apmA*. Contrary to our expectations, both *apmA* expression (Figure 5cii) and the corresponding progesterone metabolic activity (Figure S2Ci) were induced by glucose in a dose-dependent manner.

Subsequently, we determined whether *apmAB* were present in women with infertility who had gut microbiota exhibiting high progesterone metabolic activity. We extracted the total bacterial DNA from fresh fecal samples to determine the abundance of *apmA* and *apmB* in individual fecal samples. The quantitative polymerase chain reaction (PCR) results revealed a significantly higher abundance of both *apmA* (Figure 2(b); left panel) and *apmB* (Figure 2(b); right panel) in the gut microbiota with high progesterone metabolic activity, especially in the microbiota from Patients 1, 11, and 13 (Table 4). Together, these data support the role of ApmAB from *C. innocuum* in the gut progesterone metabolism of infertile patients, with epipregnanolone as the sole microbial product.

**Table 4.** Abundance of the progesterone metabolism genes *apmAB* in individual fecal samples through qPCR.

Patient no.	Progesterone metabolic activity*	*apmA* abundance	*apmB* abundance
Case 1#	active	1.56 × 10^7^	2.56 × 10^7^
Case 2	inactive	0.47 × 10^7^	0.9 × 10^7^
Case 3	inactive	0.002 × 10^7^	0
Case 4	inactive	0	0
Case 5	inactive	0.003 × 10^7^	0
Case 6	inactive	0.04 × 10^7^	0.04 × 10^7^
Case 7	active	0	0
Case 8	inactive	0.01 × 10^7^	0
Case 9	inactive	0.12 × 10^7^	0.17 × 10^7^
Case 10	active	0.13 × 10^7^	0.24 × 10^7^
Case 11	active	4.05 × 10^7^	5.8 × 10^7^
Case 12	inactive	0.08 × 10^7^	0.23 × 10^7^
Case 13	active	4.35 × 10^7^	6.13 × 10^7^
Case 14	active	0.05 × 10^7^	0.099 × 10^7^

#, *C. innocuum* strain RGG8 was isolated from the gut microbiota of Patient 1.

*Progesterone metabolic activity was determined by quantifying residual progesterone remaining in fecal cultures that were anaerobically incubated with progesterone (1 mm) in DCB-1 medium for 7 days. “Active” is represented by microbial utilization of >50% of progesterone molecules.

### *Effect of* C. innocuum *on circulating progesterone levels, the estrous cycle, and follicle development in female mice*

**Figure 6.**
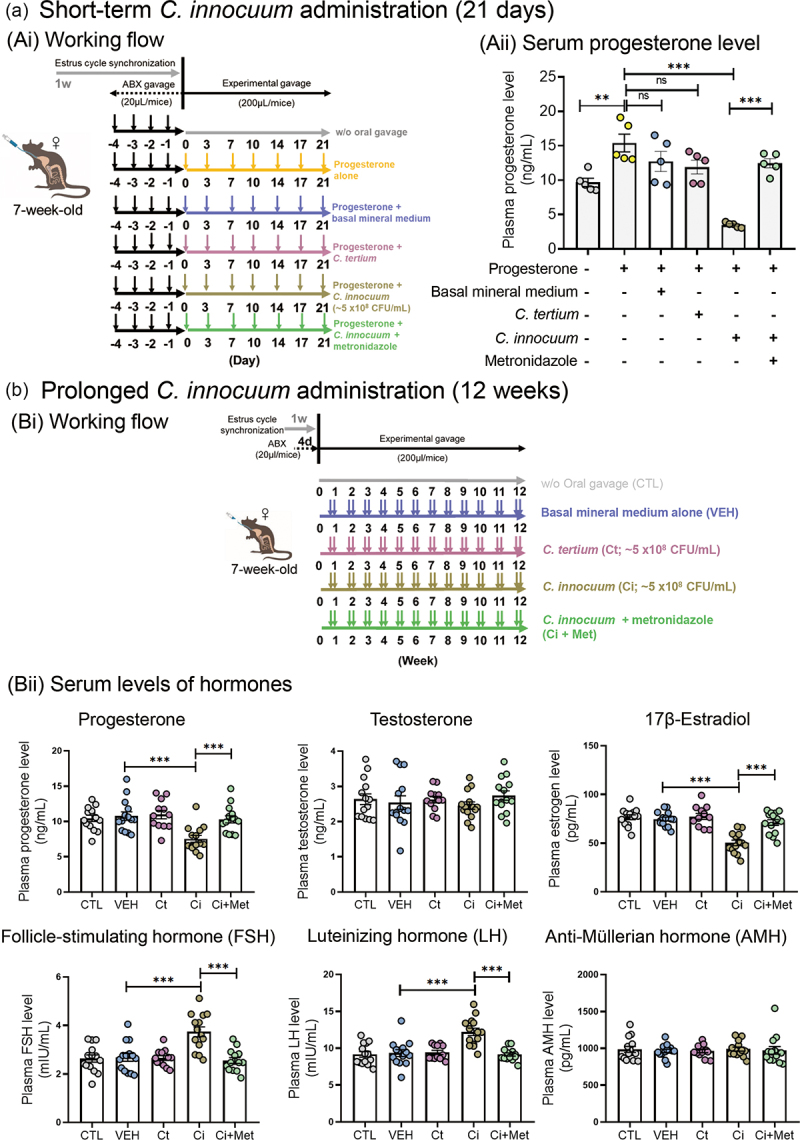
Administration of the progesterone-metabolizing *C. innocuum* strain RGG8 into the guts of female mice through oral gavage led to a decrease in host circulating progesterone levels. (a) Effects of *C. innocuum* on the bioavailability of oral progesterone supplementation. (Ai) Working flow of the administration of the strain RGG8 and progesterone in female mice through oral gavage for 21 days. (Aii) Administration of the strain RGG8 significantly reduced plasma progesterone levels in female mice treated with progesterone. (b) Long-term effects of *C. innocuum* on the plasma levels of hormones. (Ai) Working flow of the oral administration of the strain RGG8 for 12 weeks. (Bii) Administering *C. innocuum* strain RGG8 significantly reduced the levels of mouse plasma estradiol but did not affect the levels of plasma testosterone. It also increased the plasma levels of FSH and LH but did not affect the plasma levels of AMH. Statistical results were calculated using the unpaired nonparametric *t*-test; **p < 0.01, ***p < 0.001.

The present experiments suggested that *C. innocuum* may be a causal factor contributing to the low bioavailability of orally administered progesterone. We performed animal experiments using C57BL/6 mice as the animal model and investigated the effects of *C. innocuum* on the bioavailability of oral progesterone supplementation. Except for negative controls (without oral gavage; *n* = 5), all the tested mice were orally administered with progesterone (20 mg/kg/day). Female mice were administered with the strain RGG8 through oral gavage for 21 days (Figure 6ai), and plasma progesterone levels were subsequently determined through an enzyme-linked immunosorbent assay (ELISA). Compared with those of the negative control group (CTL; 9.7 ± 0.6 ng/mL), the plasma progesterone levels of the progesterone-treated mice significantly increased (15.4 ± 1.3 ng/mL, *n* = 5). We used a common human pathogen, namely *C. tertium* ,^34^ as the reference *Clostridium* species (a negative control without progesterone metabolic activity). We did not observe any difference in plasma progesterone levels among the mice treated with *C. tertium* (11.9 ± 1 ng/mL; *n* = 5), the mice treated with basal minimal medium (12.7 ± 1.5 ng/mL; *n* = 5), and the mice treated with progesterone alone. By contrast, plasma progesterone levels (3.5 ± 0.2 ng/mL) significantly decreased in *C. innocuum*-treated mice (*n* = 5). However, metronidazole treatment recovered the *C. innocuum*–mediated decrease in plasma progesterone levels (12.5 ± 0.7 ng/mL; *n* = 5) (Figure 6aii). During the entire 21-day exogenous progesterone treatment period, we assessed the stage of the mouse estrous cycle through the daily cytological examination of vaginal smears. In addition, we recorded the body weight of each mouse weekly; however, we did not observe any changes in the stage of the mouse estrous cycle or body weight.

**Figure 7.**
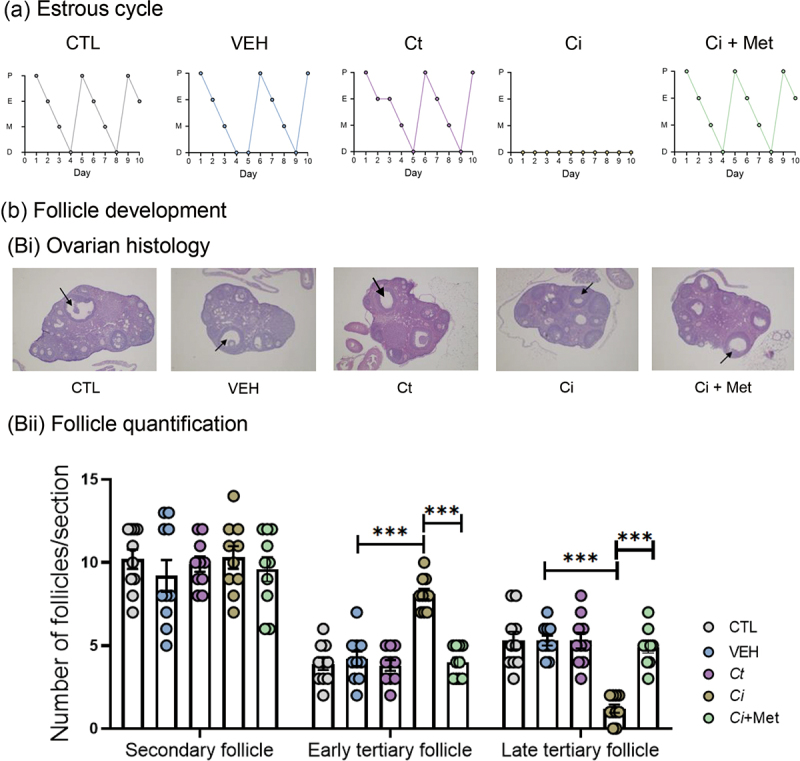
Prolonged *C. innocuum* administration (12 weeks) led to the arrest of the mouse estrous cycle (a) and follicular development (b). Data are expressed as mean ± standard error for six individual mice. Statistical results were calculated using the unpaired nonparametric *t*-test; ***p < 0.001. Abbreviations: ci, *C. innoccum* administration; ci + met, *C. innoccum* and metronidazole coadministration; ct, *C. tertium* administration; CTL, female mice without oral gavage; VEH, vehicle mice.

To investigate the long-term effects of *C. innocuum* on host reproductive physiology, we administered *C. innocuum* strain RGG8 to female mice twice per week through oral gavage for 12 consecutive weeks (Figure 6bi). The *C. tertium* was then used as a reference *Clostridium* species. Compared with the mice in the negative control group (CTL; without oral gavage; 10.4 ± 0.5 ng/mL; *n* = 10) and those in the vehicle group (VEH; with mineral medium; 10.8 ± 0.7 ng/mL; *n* = 10) and *C. tertium* treatment (Ct; 11.0 ± 0.7 ng/mL; *n* = 8), plasma progesterone levels were significantly decreased in the *C. innocuum*-treated mice (Ci; 7.5 ± 0.6 ng/mL; *n* = 10). Coadministration with metronidazole restored the decline in serum progesterone levels (Ci + Met; 10.3 ± 0.6 ng/mL; *n* = 10) (Figure 6bii). In addition, prolonged *C. innocuum* administration led to a decrease in serum estradiol levels but increases in the serum levels of follicle-stimulating hormone (FSH) and luteinizing hormone (LH) (Figure 6bii). The original levels of these hormones were restored by coadministration with metronidazole. Moreover, cytological analysis of vaginal smears demonstrated that the 12-week *C. innocuum* administration led to an arrest of the mouse estrous cycle, which was then restored by metronidazole treatment (Figure 7(a)). By contrast, the arrest of the estrous cycle was not observed among the *C. tertium*-treated mice. Furthermore, the histological analysis of the ovarian tissue revealed that compared with the CTL, VEH, and Ct (*C. tertium*-treated mice) groups, *C. innocuum* administration significantly increased the number of early tertiary follicles but decreased the number of late tertiary follicles (Figure 7(b)). Overall, our data suggest that prolonged *C. innocuum* administration leads to ovarian follicle arrest in the early antral stage, but that such arrest can be eliminated by coadministration with metronidazole.

## Discussion

To the best of our knowledge, the present study is the first study to provide robust evidence demonstrating that gut progesterone serves as an electron acceptor for the strictly anaerobic *C. innocuum* and that the respiration process occurs extracellularly and does not require progesterone uptake. Animal guts provide a highly anaerobic and reductive environment^35,36^ with abundant electron donors but few electron acceptors. Similar to quinones, the A-ring of progesterone has conjugated double bonds at C-3 and C-5, which can accept two pairs of electrons. In this study, we purified and characterized NADPH-dependent 5β-dihydroprogesterone reductase, which belongs to the Etf family. Moreover, bioinformatic analysis of 5β-dihydroprogesterone reductase (ApmAB) from *C. innocuum* revealed that this enzyme is similar to the CarDE component of caffeoyl-CoA reductase from *Acetobacterium woodii* (amino acid sequence identity = approximately 50%). In both subunits of heterodimeric 5β-dihydroprogesterone reductase, we identified a highly conserved Rossman domain for binding NADPH. Electrons are likely transferred sequentially from NADPH, FAD, and finally to progesterone, with epipregnanolone as the main product.

In a previous study, 3β-hydroxysteroid dehydrogenase was considered an alternative enzyme capable of reducing the 3-keto group in various steroids.^37^ The protein 3β-hydroxysteroid dehydrogenase [belonging to the short-chain dehydrogenase/reductase (SDR) family] is widely distributed in yeast,^38^ plants,^39^ animals,^40^ and bacteria.^41,42^ These proteins possess a highly conserved NAD(P)^+^-binding domain and are not oxygen sensitive.^39^ For example, Kenny et al. (2020)^43^ identified a NADP^+^-dependent cholesterol dehydrogenase from uncultured *Clostridium* species, which transforms cholesterol (with a 3β-hydroxyl group) into cholestenone (with a 3-keto group) in the human gut. In addition, NAD^+^-dependent 3β-hydroxysteroid dehydrogenase was purified from the cytoplasm of *C. innocuum*.^37^ This monomeric enzyme was able to mediate the reverse reaction (reduction of the 3-ketosteroids into 3β-hydroxysteroids), in which NADH was used as an electron donor. However, the enzymatic activity is very low (specific activity = 16 nmol progesterone consumed/hour/mg protein) and was not physiologically relevant. Moreover, the oxygen tolerance of the SDR-type 3β-hydroxysteroid dehydrogenase was inconsistent with the results of the present resting cell assay, which revealed the oxygen sensitivity of progesterone-metabolizing enzymes in *C. innocuum*. Accordingly, in *C. innocuum*, NAD^+^-dependent 3β-hydroxysteroid dehydrogenase may catalyze the oxidation of 3β-hydroxysteroids in the cytoplasm, and 5β-dihydroprogesterone reductase (ApmAB) may catalyze the 3-keto reduction of 3-ketosteroids on the cell membrane.

Steroids with a pregnane skeleton are effective modulators of neuronal γ-aminobutyric acid (GABA) receptors.^44^ The spatial arrangement of the hydroxyl group at the C-3 position of the steroid skeleton markedly affects the biological properties of steroids. Pregnanolone and epipregnanolone are stereoisomers of allopregnanolone, and they differ from allopregnanolone in terms of the orientations of the H-5 and 3-hydroxy groups. 3α-Hydroxypregnanes (e.g., allopregnanolone) are strong positive modulators, and in nanomolar concentrations, 3α-hydroxypregnanes can potentiate the GABA-induced chloride current; 3β-hydroxypregnanes (e.g., epipregnanolone) exhibit antagonistic properties and weaken the stimulating effect of allopregnanolone on the GABA-induced chloride current.^45^ Allopregnanolone is considered a neuroprotective agent.^4,5,46^ Other neurosteroids have not yet attracted intensive research attention. Some studies have demonstrated that epipregnanolone may have therapeutic effects that extend beyond the modulation of GABA receptors. For example, O’Dell et al. (2005)^47^ described a significant reduction of alcohol self-administration following pre-treatment with epipregnanolone, and they proposed that this effect may be related to epipregnanolone’s actions on the GABA receptor. However, contrasting results have been obtained regarding the effects of 3-hydroxypregnanes on GABA receptors, and some investigators have described epipregnanolone as a positive modulator of GABA receptor.^48^

In the present study, we observed that patients with high progesterone metabolic activity in their fecal samples exhibited low circulating progesterone levels, and we also observed the unusual capability of *C. innocuum* to decrease circulating progesterone levels, likely through the inactivation of endogenous and exogenous progesterone in the mouse gut. Thus, we argue that *C. innocuum* is a causal factor of progesterone resistance in women with infertility who take progesterone. Accordingly, individualized progesterone dosage could be determined based on the progesterone metabolism capability of the gut microbiota of a patient; such determination could not only reduce costs but also minimize the side effects associated with high-dose medications, thereby enhancing treatment efficiency. This study also observed the arrest of the follicular development and estrous cycle in mice with prolonged *C. innocuum* administration. In humans, progesterone is mainly produced by the corpus luteum in the ovaries.^49^ Follicular arrest may lead to the dysfunction of the corpus luteum, which further hinders endogenous progesterone production. Therefore, microbial activities and host follicular arrest (through an unidentified route) may lead to lower progesterone levels in both female mice and women with infertility who have high *C. innocuum* abundance. In addition to progesterone, prolonged *C. innocuum* administration led to marked changes in plasma levels of estradiol, FSH, and LH in this study. These findings imply a potential role of *C. innocuum* in human ovulatory disorders. Nonetheless, the effects of *C. innocuum* on pregnancy, miscarriage risk, and related disorders resulting from progesterone metabolic abnormalities remain to be investigated through clinical trials with a larger sample size.

## Conclusions

Members of *Clostridium*, including uncultured species, adopt hydroxysteroid dehydrogenases to metabolize cholesterol^43^ and cortisol^50^ in host guts. In this study, we identified *C. innocuum* as a major microbe that utilizes progesterone in the gut. This microbial process occurring in the gut can significantly decrease the host circulating progesterone levels through enterohepatic circulation. The biotransformation of progesterone into epipregnanolone in the host gut hinders not only the progestogenic activity of progesterone but also intestinal progestogen reabsorption, resulting in decreased circulating progesterone levels in the host. In the present study, microbial progesterone metabolism was investigated using gut microbiota collected from 14 patients with infertility. Clinical investigations with a larger population size are required to elucidate the effects of the gut microbe *C. innocuum* on human physiology and reproduction. Unfortunately, large-scale (>100 patients) gut microbiome data regarding infertile patients with oral progesterone treatment are not currently available. Nonetheless, our findings provide promising therapeutic targets for the clinical amelioration of low bioavailability of progesterone. For example, treatment with antibiotics (e.g., *Clostridium*-specific metronidazole) can be used to eliminate the gut *C. innocuum* population before oral progesterone supplementation. Moreover, the genes corresponding to 5β-dihydroprogesterone reductase are prevalent in *C. innocuum* but not identified in any other *Clostridium* species. Thus, these highly specific functional genes, especially *apmA*, can be used as clinical biomarkers for identifying patients with infertility who have low serum progesterone levels and low bioavailability of oral progesterone supplementation.

In the progesterone-treated fecal cultures, we identified at least three major products (epipregnanolone, isopregnanolone, and pregnanolone) derived from progesterone. As *C. innocuum* exclusively produce epipregnanolone, our data do not exclude the involvement of other bacterial species in gut progesterone metabolism. To the best of our knowledge, this study is the first study to provide strong evidence demonstrating that gut microbes can produce neurosteroids from progesterone. Progesterone-derived compounds with 3-hydroxyl groups are potential neurosteroids.^3^ Currently, most progesterone-derived neurosteroids are either extremely expensive or not available commercially, which considerably impedes detailed investigations of the physiological and psychological effects as well as the clinical applications of these neurosteroids. Compared with the production of neurosteroids through organic chemical approaches, the use of gut microbes (e.g., *C. innocuum*) and their enzymes may result in the production of neurosteroids with relatively few stereoisomers and regioisomers; this could considerably decrease the cost of chromatographic purification. Progesterone-transforming enzymes with high regio- and stereo-specificity thus have many biotechnological applications.

## Materials and Methods

### Participants and protocols of hormone treatment and sample collection

A total of 14 women with infertility undergoing hormone treatment for endometrial preparation for frozen–thawed embryo transfer were included in this study. To prime the endometrium, exogenous estrogen and progesterone were administered. Hormone replacement therapy was initiated on day 3 of the menstrual cycle, with estradiol valerate administered at 4 mg/day from day 3 to 8, at 8 mg/day from day 9 to 11, and at 12 mg/day from day 12 until an optimal endometrial thickness was achieved for embryo transfer. Starting from the day on which the embryo transfer procedure was scheduled, 8% progesterone gel (Crinone; Merck) was transvaginally administered at a dosage of 90 mg/day for 2 days, followed by an increased dosage of 180 mg/day for the following 14 days. Oral progesterone (Utrogestan) was also initiated at a dosage of 600 mg/day on the day of embryo transfer. All participants were required to provide fecal samples on the day preceding the initiation of progesterone treatment. Serum samples were collected three to four times on the day preceding the initiation of progesterone treatment, 2 days after the initiation of oral progesterone, and 1 week later. In case of a positive pregnancy test result, an additional blood sample was collected 1 week after the confirmation of pregnancy. This procedure was approved by the local ethics committee (Clinical Trial/Research Approval NTUH-REC no.:202103046RINB).

### Chemicals

Progesterone was purchased from Sigma-Aldrich (St. Louis, MO, USA). The other progestogens, including 5α-dihydroprogesterone, 5β-dihydroprogesterone, 3α-hydroxy-5α-pregnan-20-one (allopregnanolone), 3β-hydroxy-5α-pregnan-20-one (isopregnanolone), 3β-hydroxy-pregnan-20-one, 3α-hydroxy-5β-pregnan-20-one (pregnanolone), 3β-hydroxy-5β-pregnan-20-one (epipregnanolone), 3α-hydroxy-4-pregnen-20-one, 3β-hydroxy-4-pregnen-20-one, 20α-dihydroprogesterone, and 20β-dihydroprogesterone were purchased from Steraloids (Newport, RI, USA). Other chemicals used were of analytical grade and were purchased from Mallinckrodt Baker (Phillipsburg, NJ, USA), Merck Millipore (Burlington, VT, USA), and Sigma-Aldrich, unless otherwise specified.

### Identification of microbial progestogenic metabolites and determination of progestogenic activity in fecal cultures

**Figure 1.**
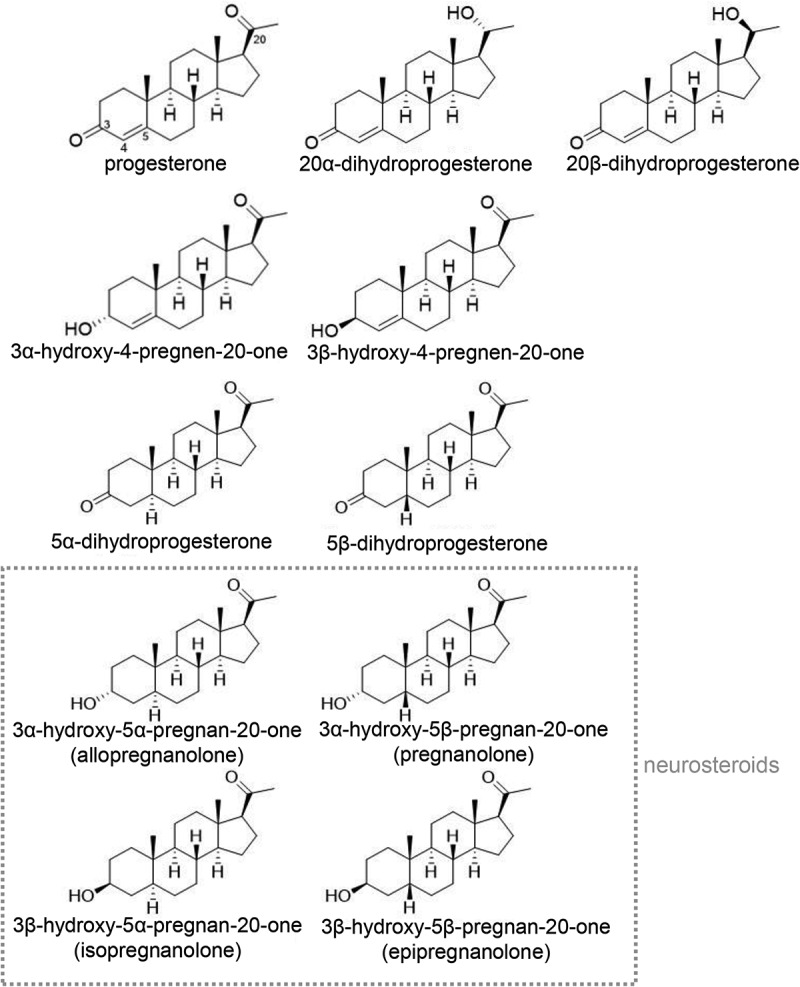
Chemical structures of common progestogens. Among these progestogens, pregnanolone, allopregnanolone, epipregnanolone, and isopregnanolone are regarded as neurosteroids. Critical carbon atoms in the steroidal numbering system are depicted on progesterone.

Fresh fecal samples were collected from 14 infertile females 20–40 (refer to Table 1 for detailed information) who underwent oral progesterone therapy for endometrial preparation and thawed embryo transfer. The samples (approximately 0.5 g each) were anaerobically incubated with progesterone (1 mm) in either a chemically defined medium with mineral salts (DCB-1, 100 mL) or Brain Heart Infusion (BHI) medium (100 mL) at 37°C in the dark. Subsequently,17α-ethinylestradiol (final concentration: 50 μM), which cannot be utilized by the gut microbiota, was added to the fecal cultures to serve as an internal control. Samples (3 mL) were extracted daily from the progesterone-enriched fecal cultures, and progesterone-derived microbial products were isolated from these culture samples (1 mL) through dual extraction with ethyl acetate. After the solvent was completely evaporated, the residue was re-dissolved in 50 μL of methanol, and progestogenic metabolites were identified using thin-layer chromatography (TLC) and ultraperformance liquid chromatography–atmosphere pressure chemical ionization–high-resolution mass spectrometry (UPLC–APCI–HRMS). Subsequently, 10 progestogens (refer to Figure 1 for individual structures), reduced at the 3-keto, 20-keto, and C-5 groups, were used as authentic standards (refer to Table S1 for the UPLC–HRMS patterns of individual progestogens). To determine the progestogenic activity of the fecal culture samples, the samples (1 mL) were extracted three times with ethyl acetate. After the solvent was completely evaporated, the residue was re-dissolved in ddH_2_O to determine its progestogenic activity (see subsequently). To determine temporal changes in the bacterial community structures of the fecal cultures, bacterial cells were collected through centrifugation (10,000 × *g* for 10 min at 4°C), and bacterial DNA was extracted from the pellet by using a QIAamp PowerFecal Pro DNA kit (Qiagen, Hilden, Germany) in accordance with the manufacturer’s instructions. Subsequently, the concentration of DNA was determined using either a NanoDrop ND-1000 spectrophotometer or a Qubit dsDNA Assay kit (Invitrogen, Thermo Fisher Scientific, Waltham, MA, USA). Bacterial 16S rRNA was amplified through a PCR process, and the resulting amplicons were sequenced using the PacBio platform (see subsequently). To investigate the effects of antibiotics on microbial progesterone metabolism, a fecal sample (approximately 0.5 g) obtained from Patient 1 was anaerobically incubated with progesterone (1 mm) and individual antibiotics in BHI broth (100 mL) at 37°C in the dark. Samples (3 mL) were extracted daily from the progesterone-enriched fecal cultures.

### Analytical chemical methods

(a) TLC

Progestogenic standards and ethyl acetate-extractable compounds were separated on a silica gel-coated aluminum high-performance TLC plate (Silica gel 60 F_254_; thickness: 0.2 mm; dimensions: 20 × 20 cm; Merck Millipore). The mobile phase consisted of dichloromethane, ethyl acetate, and ethanol (14:4:0.05, v/v/v). The compounds were visualized either by using ultraviolet light at 254 nm or by spraying the TLC plates with 30% (v/v) H_2_SO_4_, followed by heating on a hot plate.

(b) UPLC–HRMS

Progestogenic metabolites were detected using UPLC–HRMS on a UPLC system coupled to an APCI–MS system. These metabolites were separated using a reversed-phase C_18_ column (ACQUITY UPLC BEH C_18_; 1.7 μm, 100 × 2.1 mm; Waters, Milford, MA, USA) at a flow rate of 0.45 mL/min at 50°C (oven temperature). The mobile phase consisted of solution A (0.1% formic acid [v/v] in 2% acetonitrile [v/v]) and solution B (0.1% formic acid [v/v] in acetonitrile [v/v]). Separation was conducted by gradually increasing the concentration of solvent B from 40% to 55% in 9 min and then up to 100% B within 1 min. Mass spectrometric data were acquired in positive ionization mode (parent scan range: 100–500 *m*/*z*). The temperatures of the capillary and APCI vaporizer were set to 120°C and 395°C, respectively, and the sheath, auxiliary, and sweep gas flow rates were adjusted to 40, 5, and 2 arbitrary units, respectively. The source voltage was maintained at 6 kV, and the current was adjusted to 15 μA. The elemental composition of individual adduct ions was predicted using Xcalibur Software (Thermo Fisher Scientific).

### Isolation of progesterone-metabolizing gut microbes through a culturomic approach

Progesterone-metabolizing bacteria were isolated from human fecal samples by using culturomics. Briefly, fecal samples (5 g) were suspended in anaerobic DCB-1 broth (100 mL) and shaken vigorously. The resulting suspensions were serially diluted (up to 10^−6^), after which 100 uL aliquots were collected from the 10^−3^ to 10^−6^ dilutions and spread on agar plates designed for various bacterial taxa. These plates were subsequently incubated in an anaerobic chamber (Coy Laboratory Products, Grass Lake, MI, USA). Gifu anaerobic medium (GAM; BD-Difco, Sparks, MD, USA) was used to isolate gut anaerobes, including those belonging to the Family Clostridiales. *Clostridium butyricum* isolation medium (BIM)^51^ and iron sulfite agar (HiMedia, Mumbai, India) were used to isolate *C. butyricum* and other related species. *Bacteroides Fragilis* selective medium (BFS)^52^ was used to isolate members of the phylum Bacteroidetes. Raffinose-*Bifidobacterium* (RB) medium^53^ and BFM selective medium^54^ were used to isolate Bifidobacteria and related taxa. De Man–Rogosa–Sharpe medium (BD-Difco) was used to isolate lactic acid bacteria and other related taxa. Progesterone (1 mm) was added to all growth media. Anaerobic growth media were prepared using the Hungate technique, and the bacterial cultures were grown on agar in an anaerobic chamber. All glassware, media, petri dishes, and reagents were maintained under anaerobic conditions. After anaerobic incubation for 2–5 days at 37°C, colonies were observed, and individual colonies were selected and inoculated in 800 μL of GAM broth in 48-well plates. These plates were incubated at 37°C in an anaerobic chamber with agitation (100rpm) for 3 days. Active cultures (OD_600 nm_ > 0.1) were used as pre-cultures for subsequent high-throughput screening, as described subsequently.

### High-throughput screening for anaerobic progesterone metabolic activity

Bacterial colonies were screened for anaerobic progesterone metabolic activity by transferring 100 μL of the pre-cultures to fresh GAM broth in 24-well plates, each containing 1 mm progesterone. The plates were subsequently incubated at 37°C with agitation (100 rpm) for 24–48 h in an anaerobic chamber. Next, microbial activity was monitored using high-throughput TLC. Active bacterial isolates were preserved in the GAM broth supplemented with 20% glycerol or 5% dimethyl sulfoxide (DMSO), and the resulting axenic cultures were stored at −80°C.

### Anaerobic growth of strain RGG8 on progesterone

The strain RGG8 was routinely cultured in the BHI broth (100 mL) supplemented with progesterone (1 mm). 17α-Ethinylestradiol (50 μM, indigestible by strain RGG8) was added as an internal control. Anaerobic cultures were incubated in the dark at 37°C with stirring (approximately 180 rpm), and cultural samples (3 mL) were collected every 12 h. After the progesterone-derived metabolites extracted from these samples were identified and quantified using UPLC–HRMS, the progestogenic activity of the cultural samples was evaluated using a yeast progestogenic activity assay, as described subsequently. To evaluate bacterial growth (represented as an increase in protein content), culture samples of the strain RGG8 were centrifuged at 10,000 × *g* for 10 min. After centrifugation, the cell pellet was resuspended in 1 mL of reaction reagent (Pierce BCA protein assay kit; Thermo Fisher Scientific), and the protein content was determined using a BCA protein assay in accordance with manufacturer’s instructions, with bovine serum albumin serving as the standard.

### BioVAL A-YPS yeast progestogenic activity assay

An assay of the progestogenic activity was conducted using a BioVAL A-YPS kit in accordance with the manufacturer’s instructions. In the A-YPS kit, the non-conventional and recombinant yeast *Arxula adeninivorans* is used as a progesterone-responsive biosensor, and the reporter protein, phytase, is directly secreted into the medium. A positive correlation exists between phytase production and progestogenic activity; hence, phytase can be measured through the spectrophotometric monitoring of the enzymatic reaction with a chromogenic substrate. In this study, individual progestogenic standards or dried ethyl acetate extracts were dissolved in and diluted using ddH_2_O. The resulting aqueous solutions (400 μL) were added to yeast cultures (100 μL, initial OD_600 nm_ = 0.5) on a 96-well microtiter plate. Subsequently, the bioassay mixtures were incubated at 30°C for 24 h. After incubation, substrate buffer (50 μL) from the kit was added to each well of the 96-well plate, and the mixtures were further incubated at 37°C for 30 min. The reaction was stopped by adding 100 μL of developer solution to the bioassay mixtures, and the absorption of the mixtures was measured at a wavelength of 405 nm using a microtiter plate spectrophotometer.

### Preparation of cell extracts of strain RGG8

*C. innocuum* strain RGG8 was anaerobically cultivated in BHI broth (total volume: 10 L) supplemented with 50 μM progesterone as an inducer. The bacterial cells were harvested at an OD_600 nm_ value of approximately 0.8 through centrifugation. The cell pellet (10 g) was then re-suspended in 30 mL of suspension buffer (pH 8.5) containing Tris-HCl (50 mm), 2-mercaptoethanol (10 mm), cOmplete mini-protease inhibitor, glycerol (5%), and DNase I (1 U). Subsequently, strain RGG8 cells were broken by passing the cell suspension through a French pressure cell (Thermo Fisher Scientific) twice at 137 megapascals in an O_2_-free system. The cell-free lysates were fractionated using two steps of centrifugation. First, the cell-free lysates were centrifuged at 20,000 × *g* for 30 min to remove most cell debris and unbroken cells. Second, the supernatants containing the crude cell extracts were centrifuged at 100,000 × *g* for 1.5 h to isolate soluble proteins from membrane-bound proteins. Before ultra-centrifugation, the cell-free lysates were treated with 0.2% (w/v) Tween 20, a mild detergent, to solubilize peripheral membrane proteins. All procedures for the preparation of cell extracts were conducted at 4°C.

### Purification of ApmAB from crude cell extracts of strain RGG8

The crude cell extracts were precipitated at 30% (v/v) ammonium sulfate saturation. After centrifugation, the pellet was re-dissolved in buffer A (50 mm Tris–HCl and 10 mm 2-mercaptoethanol, pH 8.5). Subsequently, the salts were removed using a prepacked PD-10 desalting column (8.3 mL; Amersham Biosciences Europa, Freiburg, Germany) in accordance with the manufacturer’s instructions. Further protein purification was conducted using fast protein liquid chromatography (FPLC) within an anaerobic chamber, and bacterial proteins were sequentially separated using diethylaminoethyl (DEAE)-Sepharose (ion exchange), Phenyl-Sepharose (hydrophobic interaction), and Sephacryl S-300 (gel filtration). Briefly, the bacterial proteins were introduced into a DEAE-Sepharose column (5 mL) that had been pre-equilibrated with buffer A (50 mm Tris–HCl, pH 8.5). Subsequently, elution was performed using buffer B (50 mm Tris–HCl, 1 M KCl, and 10 mm 2-mercaptoethanol, pH 8.5), with a gradient of 0% to 50% within 30 min at a flow rate of 1 mL/min. The activity of ApmAB was determined as described subsequently. Active protein fractions were collected and introduced into a Phenyl-Sepharose column (1 mL; GE Healthcare, Chicago, IL, USA) equilibrated with buffer B; elution was then performed using buffer A with a gradient from 0% to 100% within 1 h at a flow rate of 0.5 mL/min. Subsequently, active fractions were pooled and concentrated using a Vivaspin 500 centrifugal concentrator. The resulting proteins were introduced into a TSKgel G3000SW HPLC Column equilibrated with buffer A (50 mm Tris–HCl, 100 mm KCl, and 10 mm 2-mercaptoethanol, pH 8.5) at a flow rate of 0.5 mL/min. Active fractions were also pooled and concentrated using a Vivaspin 500 centrifugal concentrator.

### Resting cell and in vitro enzymatic assays

Resting cell and *in vitro* enzymatic assays were routinely conducted in the dark at 30°C under oxic or anoxic conditions for 1.5 h. For the resting cell assay, *C. innocuum* strain RGG8 and *Eubacterium limosum* (reference strain) cells were harvested during the middle log phase (with an OD_600 nm_ of approximately 0.8) through centrifugation, and the pellets were washed twice with Tris-HCl buffer (50 mm, pH 8.5). The assay mixtures (0.5 mL) contained Tris-HCl buffer (50 mm, pH 8.5), cell pellets (0.1 g), progesterone (0.2 mm), and 2-mercaptoethanol (10 mm). In some assays, Ti(III) citrate (3 mm), methyl viologen (0.5 mm), and an ATPase inhibitor (sodium orthovanadate, 10 mm) were added to the mixtures. For the *in vitro* enzymatic assay, the assay mixtures (0.5 mL) contained Tris-HCl buffer (50 mm, pH 8.5), crude cell extract (or protein fractions) from the strain RGG8, 5β-dihydroprogesterone (substrate for ApmAB, 0.1 mm), NADPH (20 mm), and 2-mercaptoethanol (10 mm). After 1.5 h of anaerobic incubation, the mixtures were extracted twice with the same volume of ethyl acetate. Subsequently, the ethyl acetate extracts were pooled, vacuum-dried, and stored at −20°C for further analysis. TLC and UPLC–HRMS were used to monitor the production of epipregnanolone.

### General molecular biological methods

Bacterial genomic DNA, including that of the strain RGG8, was extracted using a Presto Mini gDNA Bacteria Kit (Geneaid, New Taipei City, Taiwan). The PCR mixtures (50 μL) contained nuclease-free H_2_O, 2 × PCR master mix (Invitrogen Platinum Hot Start PCR 2X Master Mix; Thermo Fisher Scientific), forward and reverse primers (200 nM each), and template DNA (10–30 ng). The PCR products were verified using standard TAE-agarose gel (1.5%) electrophoresis with SYBR Green I nucleic acid gel stain (Invitrogen, Thermo Fisher Scientific); they were then purified using a GenepHlow Gel/PCR Kit (Geneaid). TA cloning was performed using a T&A Cloning Vector Kit (Yeastern Biotech, New Taipei City, Taiwan).

### PacBio sequencing of strain RGG8 genome (accession no.: CP150439)

For library preparation and PacBio sequencing, approximately 1 μg of strain RGG8 genomic DNA was sheared using a Covaris g-TUBE (Covaris, Woburn, MA, USA) and purified using AMPure PB beads (PacBio, Menlo Park, CA, USA). The sheared and purified DNA fragments were employed as templates to prepare an SMRTbell library by using SMRTbell Template Prep Kit 1.0 (PacBio), in accordance with the manufacturer’s instructions. After adaptor ligation, inserts with a suitable size for sequencing were selected using a BluePippin system. SMRT sequencing was subsequently performed on SMRT 1 M Cell v3 (PacBio) with Chemistry version 3.0 on a PacBio Sequel sequencer (Genomics BioSci & Tech, Taipei, Taiwan). Primary filtering analysis was conducted using a Sequel System, and secondary analysis was conducted using SMRT analysis pipeline version 8.0. For genome assembly, filtered subreads from SMRT Link v8.0 were assembled using the long-read assembly algorithm Flye v2.7. SSPACE-LongRead v1.1 was applied for draft genome scaffolding, and PBJelly v15.8.24 was used for gap closure. Subsequently, genome polishing was performed using Arrow v2.3.3 software (PacBio). Further assembly of the circularizing genome was conducted using Circlator v1.5.5. Finally, the quality of the assembled genome was evaluated using QUAST v4.5. After the *de novo* genome assembly, the annotations of genomic bacterial features were determined using Prokka v1.13.

### Phylogenetic analysis of ApmAB

To elucidate the phylogenetic relationships between 5β-dihydroprogesterone reductase (ApmAB) and other Etfαβ sequences, we constructed a maximum likelihood (ML) phylogenetic tree by using 356 concatenated Etfαβ-like sequences. These sequences originated from two primary sources: 205 ApmAB-like sequences derived from publicly available genomes of *C. innocuum* and 150 Etfαβ sequences representative of five distinct groups, as identified in a foundational study on the systematic classification of Etfαβ sequences.^33^ Multiple protein sequence alignments were performed using MUSCLE version 5.1; its default algorithm was used for the alignments. The ML phylogenetic tree was created using the Le_Gascuel_2008 model, with a discrete Gamma distribution being incorporated to account for evolutionary rate differences among sites. Bootstrap values were calculated from 500 replicates to evaluate the robustness of the tree. Dataset S2 provides comprehensive details on all genome accession numbers, the phylogeny of the analyzed genomes, and the locus tags for Etfα- and β-like genes.

### *Quantification of* apmAB *in fecal samples through quantitative PCR*

The copy number of *apmAB* in fecal DNA samples was determined using quantitative PCR (qPCR), as described in a previous study.^17^ Calibration curves were obtained through a 10-fold serial dilution of the full-length PCR products of *C. innocuum apmA* and *apmB* (Figure S2Cii). Table S2 lists the primer sets used for full-length *apmAB* cloning and qPCR.

### Determination of the expression of strain RGG8 genes under different growth conditions

RNA was extracted from RGG8 cells treated with different chemicals (e.g., quinoline, menadione, progesterone, and glucose) by using a Direct-zol RNA Miniprep kit (Zymo Research, Irvine, CA, USA), and complementary DNA was generated using a SuperScript IV First-Strand Synthesis System (Thermo Fisher Scientific), as described in a previous study.^17^ The relative expression levels of *apmA* under different treatment conditions were evaluated using the 2^−ΔΔCt^ method, with the Ct value of bacterial universal 16S rRNA (refer to Table S2 for nucleotide sequences) serving as the internal control. The expression levels of *apmA* in the control groups (treated with DMSO or 0.05% glucose) were set as 1.

### PacBio sequencing of bacterial 16S rRNA gene amplicons

Genomic DNA from bacteria was extracted using a column-based kit (QIAamp PowerFecal DNA Kit; Qiagen). Subsequently, the concentration of DNA was determined using a Qubit 4.0 Fluorometer (Thermo Scientific). The entire bacterial 16S rRNA gene (regions V1–V9) was amplified using a barcoded 16S gene-specific universal primer set. On the basis of the PacBio standard protocol for the amplification of a full-length 16S gene with barcoded primers for multiplexed SMRTbell library preparation and sequencing, each primer was designed to include a 5′ buffer sequence (GCATC) with a 5′ phosphate modification, a 16-base barcode, and degenerate 16S gene-specific universal forward or reverse primer sequences (forward: 5′Phos/GCATC–16-base barcode – AGRGTTYGATYMTGGCTCAG-3′; reverse: 5′Phos/GCATC–16-base barcode – RGYTACCTTGTTACGACTT-3′); the corresponding degenerate base identities were as follows: *M* = A, C; *R* = A, G; and Y = C, T. Briefly, 2 ng of gDNA was used for a PCR process conducted using KAPA HiFi HotStart ReadyMix (Roche) under the following conditions: initial denaturation at 95°C for 3 min; followed by 20–30 cycles (sample dependence) at 95°C for 30 s, 57°C for 30 s, and 72°C for 60 s; and a final elongation step for 5 min at 72°C, followed by incubated at 4°C. The success of each PCR amplification was examined on agarose gel (1%). Samples with a bright band around 1,500 bp were selected and purified using the AMPure PB Beads for the downstream PacBio library preparation. The raw PacBio fastq files were deposited in the NCBI SRA under BioProject PRJNA1088978 (accession no.: SRR30505914~SRR30505967).

### Bacterial community structure analysis

Raw PacBio reads were quality filtered and analyzed using QIIME2-DADA2 pipeline version 2023.5.^55^ After low-quality sequences (i.e., sequences shorter than 1000 bp or longer than 1600 bp, including primer and chimeric sequences) were removed and dereplicated, amplicon sequence variants (ASVs) with a near-zero error rate and single-nucleotide resolution were generated. Subsequently, these ASVs were classified to the species level by comparing them against bacterial 16S rRNA RefSeq sequences from the NCBI nucleotide database (https://ftp.ncbi.nlm.nih.gov/refseq/TargetedLoci/Bacteria); ASVs with sequence similarity above 98.0% and 94.5% were classified to the same species and genus, respectively. Each sample was rarefied (i.e., normalized) to an equal sequencing depth (i.e., 10075 reads), and the number of ASVs per sample was transformed into relative abundance (i.e., proportion, %).

### The NMDS and PERMANOVA analysis of the progesterone-treated gut microbiota

Fresh fecal samples collected from a total of 14 female patients (Active group, *n* = 6; Inactive group, *n* = 8) were anaerobically incubated in the DCB-1 broth containing 1 mm of progesterone, and the fecal cultures were sampled at different incubation stages (initial stage, Day 0; early stage, Day 2; middle stage, Day 4; and late stage, Day 6). To determine the similarities between the fecal cultures, the contents of progesterone and epipregnanolone, as well as the abundance of *Clostridium* in individual cultural samples were visualized through the NMDS analysis. The dissimilarity between the cultural samples was analyzed using the PERMANOVA. The ellipses around the Active microbiota sampled at longer incubation stages (Days 4 and 6) were shown at confidence level of 60%.

### Preparation of strain RGG8 cell suspension for administration in mice

The strain RGG8 was anaerobically cultured in GAM broth (600 mL), and the derived cultures were incubated at 37°C in an orbital shaker (120 rpm) for approximately 16 h. The bacterial cells were harvested through centrifugation, and the cell pellets were resuspended in anaerobic phosphate-buffer saline (15 mL). Subsequently, the colony-forming units (CFUs) of the resulting cell suspension were determined by counting the strain RGG8 colonies grown on GAM agar. Finally, the cell suspensions of the strain RGG8 were stored at 4°C (within 5 days) before use.

### Strain RGG8 administration through oral gavage

C57BL/6J mice aged 7-weeks were obtained from the Animal Center of the College of Medicine of National Taiwan University (Taipei, Taiwan) and maintained under standard conditions in accordance with health guidelines for the care and use of experimental animals. All experiments were approved by the local ethics committee (IACUC No. 20220423). After 1 week of acclimatization – including estrous cycle synchronization with male mouse urine and microbiota removal through the administration of ABX solution (containing amphotericin-B 0.1 mg/mL, ampicillin 10 mg/mL, neomycin 10 mg/mL, metronidazole 10 mg/mL, and vancomycin 5 mg/mL) through oral gavage – mice of similar body weight (16–18 g) were randomly assigned to treatment groups (see Figures 6ai, bi). For the administration of the stain RGG8, approximately 5 × 10^8^ CFUs (suspended in 200 uL of basal mineral medium) were fed to each mouse through oral gavage twice per week. For the administration of exogenous progesterone through oral gavage, Utrogestan (micronized progesterone; 20 mg/kg/day; OLIC Thailand, Ayutthaya, Thailand) suspended in sesame oil (Sigma-Aldrich) was orally administered to the mice. Fresh fecal samples were collected daily, and body weight was measured once per week. Subsequently, the mice were euthanized after either 4 or 12 weeks (anesthetized with 3% isoflurane), and blood samples were collected through cardiac puncture. In addition, the ovaries were excised, weighed, fixed in neutral-buffered 4% formaldehyde for 24 h, washed with distilled water, dehydrated, and embedded in paraffin. Finally, serum samples were stored at −80°C before use.

### Vaginal smears for estrus phase determination

Before the mouse was euthanized, the estrous cycle stage of each mouse was determined through daily vaginal smears (performed for 10 days). These vaginal smears were performed as described in a previous study.^56^ Briefly, a small amount of saline solution was introduced into the mouse vagina with a disposable pipette. Subsequently, the solution was removed from the mouse vagina and placed on a slide. After staining using hematoxylin, the samples were examined under a microscope. The average interestrus interval was calculated as the average duration of the dominant presence of characteristic epithelial cells (non-nucleated and cornified) with a high cell density during the 10-day experimental period for vaginal smears.

### Ovarian histology and antral follicle count

The ovaries were serially sectioned on the long axis at 5-μm intervals, and every fifth section was stained with hematoxylin and eosin. The section with the largest diameter of each ovary was considered representative of that ovary. For each representative section, the total number of follicles and the number of follicles at each developmental stage were determined as described in a previous study.^57^ Follicles with intact, non-fragmented oocytes in the representative sections were counted and categorized by developmental stage, which was determined after a review of all adjacent sections containing follicles. All histopathological counts and classifications of follicles were conducted by two observers who reviewed the slides together before finalizing the counts. The two observers were blinded to the treatment group. Secondary follicles were characterized by two or more layers of granulosa cells without a visible antrum, and tertiary follicles were characterized by the presence of an antrum. Tertiary follicles were further divided into early and late tertiary follicles depending on estimated oocyte maturity. Pre-ovulatory (Graafian) follicles and follicles with diameters ≥250 μm, a measure considered to be indicative of oocyte maturity in mice, were classified as late tertiary follicles. Tertiary follicles without these developmental features were classified as early tertiary follicles. Secondary follicles were counted at 400× magnification, and tertiary follicles were counted at 40× magnification.

### Determination of blood levels of hormones

Blood progesterone levels in women with infertility were measured using indirect chemiluminescence (VITROS ECiQ; Ortho Clinical Diagnostics, Rochester, NY, USA).^58^ The mouse plasma levels of individual hormones were determined through ELISA kits in accordance with the manufacturer’s instructions. Plasma progesterone levels in mice were measured using a Crystal Chem Mouse Progesterone ELISA Kit (Crystal Chem, Elk Grove Village, IL, USA). The Plasma levels of estradiol and testosterone were determined using the Estradiol Parameter Assay Kit (R&D Systems; Minneapolis, MN, USA) and Testosterone Parameter Assay Kit (R&D Systems), respectively. The plasma levels of FSH, LH, and AMH were, respectively, determined using the Mouse FSH ELISA Kit (Cusabio Biotech, Houston, TX, USA), Mouse LH ELISA Kit (Cusabio Biotech), and the Mouse AMH ELISA Kit (Cusabio Biotech).

### Statistical analyses

All the biological experiments were repeated in at least triplicate. The probability value (*p* value) was examined by Welch’s t-test using GraphPad Prism 8.

## Supplementary Material

Supplemental Material

## Data Availability

Genomic data related to the strain RGG8 (accession no.: CP150439) are available in Dataset S1. Genes of the Etf protein family selected for phylogenetic analysis are detailed in Dataset S2. The raw reads of all 16S amplicons were deposited in the NCBI Sequence Read Archive (SRA) with the Bioproject PRJNA1088978 (accession no.: SRR30505914~SRR30505967).

## Highlights

- We identified *Clostridium innocuum* as a key player in gut progesterone metabolism.
- Progesterone is converted into epipregnanolone with negligible progestogenic activity.
- We identified the enzymes and mechanisms of microbial epipregnanolone production.
- *C. innocuum* caused decreased plasma progesterone and follicular arrest in female mice.
- *C. innocuum* is a causal factor of progesterone resistance in women taking progesterone.
