# Supplementary material for: Clostridium innocuum, an opportunistic gut pathogen, inactivates host gut progesterone and arrests ovarian follicular development
Source: Gut Microbes. 2024 Nov 7;16(1):2424911. doi: 10.1080/19490976.2024.2424911 (PMC11545266; doi:10.1080/19490976.2024.2424911)
Supplement: Supplemental Material [file KGMI_A_2424911_SM3095.zip › Supplementary file-KGMI 2424911/ID246109777_Clostridium_Progesterone_Supplemental_InformationI_R1.docx]

Supplementary Information for

*Clostridium innocuum*, an opportunistic gut pathogen, inactivates host gut progesterone and arrests ovarian follicular development

Mei-Jou Chen, Chia-Hung Chou, Tsun-Hsien Hsiao, Tien-Yu Wu, Chi-Ying Li, Yi-Lung Chen, Kuang- Han Chao, Tzong-Huei Lee, Ronnie G Gicana, Chao-Jen Shih, Guo-Jie Brandon-Mong, Yi-Li Lai, Po- Ting Li, Yu-Lin Tseng, Po-Hsiang Wang, and Yin-Ru Chiang

Correspondence to Po-Hsiang Wang and Yin-Ru Chiang

Email: [pohsiang@ncu.edu.tw](mailto:pohsiang@ncu.edu.tw) (Po-Hsiang Wang); E-mail: [yinru915@gate.sinica.edu.tw](mailto:yinru915@gate.sinica.edu.tw) (Yin-Ru Chiang)

## This PDF file includes the following:

Figures S1 to S2 Tables S1 to S2

Legends for Datasets S1 and S2

## Other supplementary materials for this manuscript include the following:

Dataset S1 Dataset S2

# Supplemental Figures


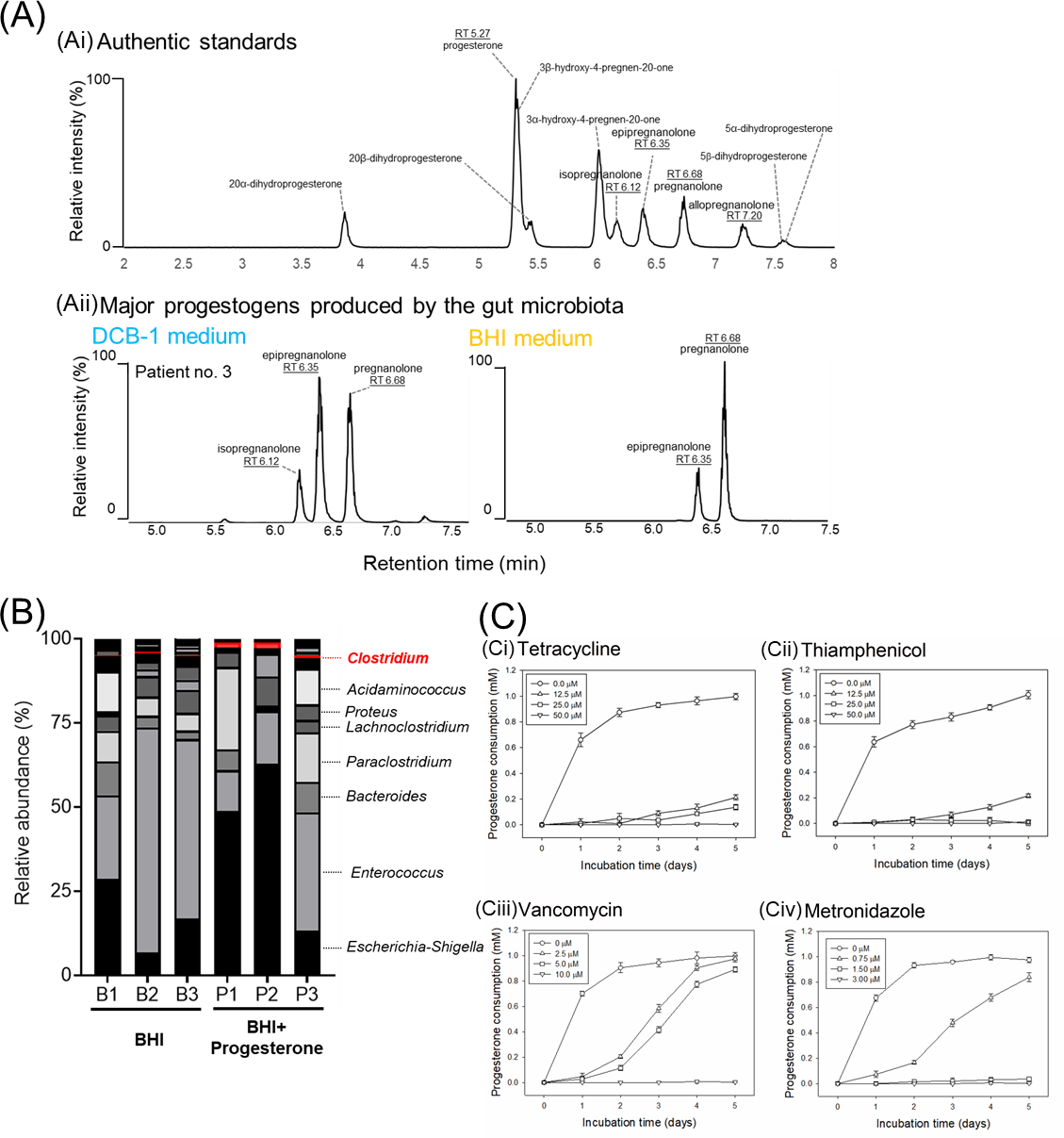


**Figure S1.** (**A**) Identification of major progestogenic metabolites produced by the gut microbiota of Patient 1. (**Ai**) UPLC chromatogram of individual progestogenic standards. (**Aii**) Major progestogenic metabolites produced by the gut microbiota. Both pregnanolone and epipregnanolone were produced in fecal cultures anaerobically incubated with progesterone (1 mM) in the DCB-1 (left panel) and BHI (right panel) media. Isopregnanolone was only detected in the DCB-1 culture. (**B**) Effect of progesterone administration on gut microbiota**.** Gut bacterial communities (from Patient 1) across different treatments (BHI broth incubated with or without 1 mM progesterone, in triplicate) were analyzed by sequencing bacterial 16 rRNA amplicons on a PacBio platform. (**C**) Effects of two broad-spectrum antibiotics, tetracycline (**Ci**) and thiamphenicol (**Cii**), and two *Clostridium*-specific antibiotics, vancomycin (**Ciii**) and metronidazole (**Civ**), on anaerobic progesterone metabolism by the gut microbiota from Patient 1.


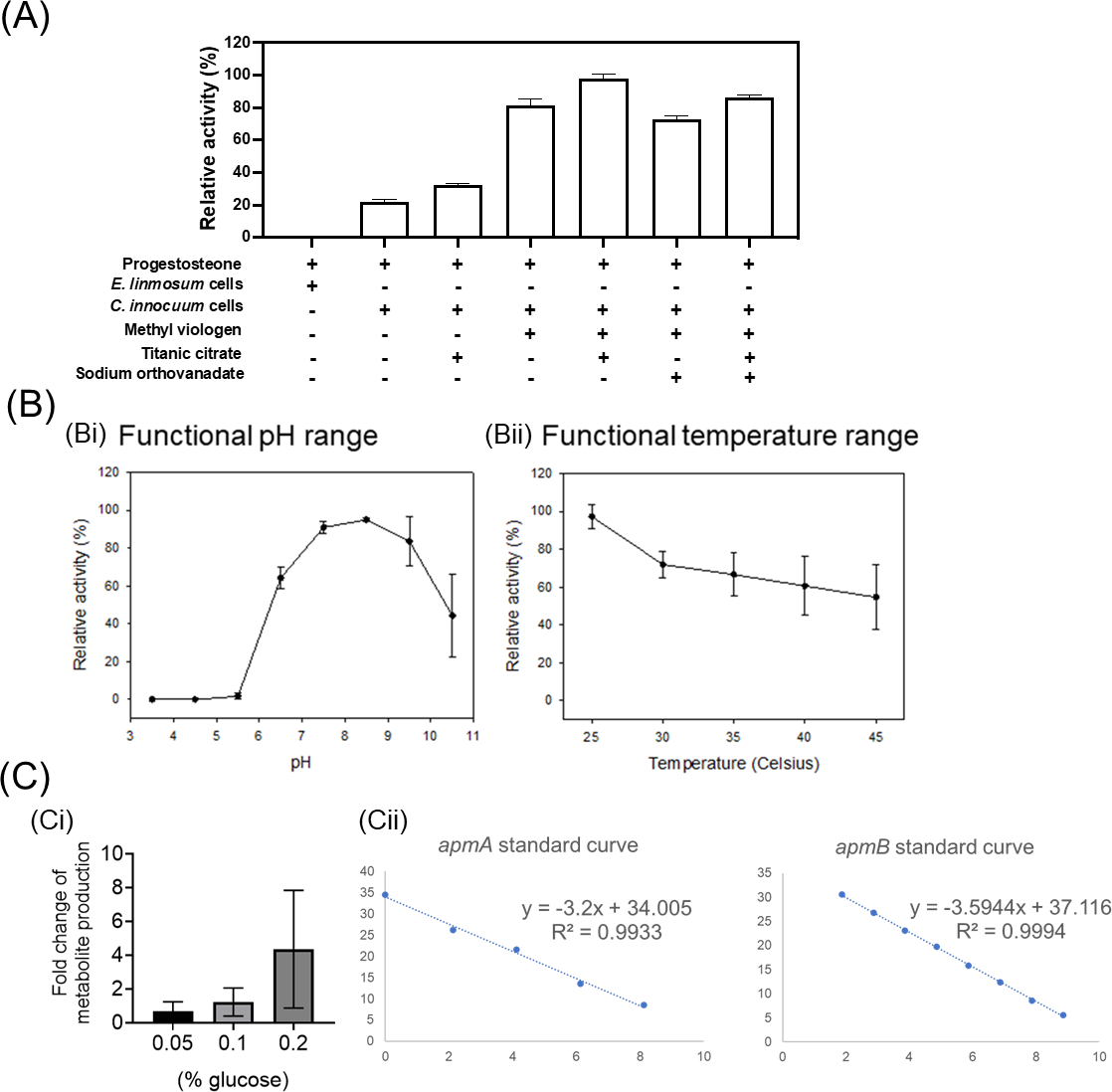


**Figure S2.** (**A**) Resting cell assays results, indicating the extracellular electron carrier requirement and oxygen sensitivity for progesterone metabolism by *C. innocuum* strain RGG8 cells. The ATPase inhibitor (sodium orthovanadate) did not reduce progesterone metabolic activity. (**B**) Optimal working pH and temperature of 5β-dihydroprogesterone reductase purified from the strain RGG8. (**C**) Expression of functional genes of the strain RGG8. (**Ci**) Effect of glucose on the production of epipregnanolone by the strain RGG8. Metabolite production was examined using thin-layer chromatography in three individual experiments. Data are presented as mean ± standard error. **(Cii)** Standard quantitative polymerase chain reaction curves of primer pairs specific for *apmA* (*R*^2^ = 0.99) and *apmB* (*R*^2^ = 1.00).

# Supplemental Tables

**Table S1.** UPLC-HRMS patterns of individual progestogenic standards.

| **Compound** | **Chemical formula** | **Molecular Weight** | **Adduct *m/z*** | **RT (min)** |
| --- | --- | --- | --- | --- |
| 5β-dihydroprogesterone | C H O  21 32 2 | 316.48 | 317.2469  +  [M+H] | 7.56 |
| 5α-dihydroprogesterone | C H O  21 32 2 | 316.48 | 317.2472  +  [M+H] | 7.59 |
| 3α-hydroxy-4-pregnen-20-one | C H O  21 32 2 | 316.48 | 299.2362  +  [M-H O+H]  2 | 6.02 |
| 3β-hydroxy-4-pregnen-20-one | C H O  21 32 2 | 316.48 | 299.2368  +  [M-H O+H]  2 | 5.31 |
| 20α-dihydroprogesterone | C H O  21 32 2 | 316.48 | 317.2468  +  [M+H] | 3.86 |
| 20β-dihydroprogesterone | C H O  21 32 2 | 316.48 | 317.2471  +  [M+H] | 5.43 |
| 3β-hydroxy-5α-pregnan-20-one (isopregnanolone) | C H O  21 34 2 | 318.49 | 301.2523  +  [M-H O+H]  2 | 6.12 |
| 3β-hydroxy-5β-pregnan-20-one (epipregnanolone) | C H O  21 34 2 | 318.49 | 301.2522  +  [M-H O+H]  2 | 6.35 |
| 3α-hydroxy-5α-pregnan-20-one (allopregnanolone) | C H O  21 34 2 | 318.49 | 301.2522  +  [M-H O+H]  2 | 7.20 |
| 3α-hydroxy-5β-pregnan-20-one (pregnanolone) | C H O  21 34 2 | 318.49 | 301.2522  +  [M-H O+H]  2 | 6.68 |
| Progesterone | C H O  21 30 2 | 314.22 | 315.2423  +  [M+H] | 5.27 |

**Table S2.** Nucleotide sequences of the PCR primers used in this study.

| Primer | Sequence (5′- 3′) | Usage | **Reference** |
| --- | --- | --- | --- |
| *apmA* full-length primer F | ATGAAAATTATCGTTCTTGTAAAAC | Amplifying *apmA* gene (full-length) from strain RGG8 DNA | This study |
| *apmA* full-length primer R | CTATTTCGTAATAATCTGCTTGTC | Amplifying *apmA* gene (full-length) from strain RGG8 DNA | This study |
| *apmA* qPCR primer F | ACATTGATTCGTGCCGGAGT | Quantifying the expression of strain RGG8-specific *apmA* | This study |
| *apmA* qPCR primer R | TTCAGCATTCCCGTAGCCTG | Quantifying the expression of strain RGG8-specific *apmA* | This study |
| *apmB* full-length primer F | ATGGCTAAATTTGAAGGATATAAA | Amplifying *apmB* gene (full-length) from strain RGG8 DNA | This study |
| *apmB* full-length primer R | TTATCCCTTTTTCGCAGC | Amplifying *apmB* gene (full-length) from strain RGG8 DNA | This study |
| *apmB* qPCR primer F | CACGTTTGAATGCCGGTCTG | Quantifying the expression of strain RGG8-specific *apmB* | This study |
| *apmB* qPCR primer R | CCATCTGCGGACGAGTATCC | Quantifying the expression of strain RGG8-specific *apmB* | This study |
| Bacteria 16s universal primer 27F | AGAGTTTGATCCTGGCTCAG | Amplifying bacteria 16s rRNA gene from total DNA extracts | Miller et al. (2013)* |
| Bacteria 16s universal primer 1492R | GGTTACCTTGTTACGACTT | Amplifying bacteria 16s rRNA gene from total DNA extracts | Miller et al. (2013) |

*Reference: Miller, C.S., Handley, K.M., Wrighton, K.C., Frischkorn, K.R., Thomas, B.C., and Banfield, J.F. (2013). Short-read assembly of full-length 16S amplicons reveals bacterial diversity in subsurface sediments. PLoS One 8, e56018.

# Legends for Datasets

**Dataset S1 (separate file).** Genome annotation of the strain RGG8.

**Dataset S2 (separate file).** Genes of Etf protein family selected for phylogenetic analysis.
